# Association Between Ambient Air Pollution and Birth Weight by Maternal Individual- and Neighborhood-Level Stressors

**DOI:** 10.1001/jamanetworkopen.2022.38174

**Published:** 2022-10-25

**Authors:** Zhongzheng Niu, Rima Habre, Thomas A. Chavez, Tingyu Yang, Brendan H. Grubbs, Sandrah P. Eckel, Kiros Berhane, Claudia M. Toledo-Corral, Jill Johnston, Genevieve F. Dunton, Deborah Lerner, Laila Al-Marayati, Fred Lurmann, Nathan Pavlovic, Shohreh F. Farzan, Theresa M. Bastain, Carrie V. Breton

**Affiliations:** 1 Department of Population and Public Health Sciences, Keck School of Medicine, University of Southern California, Los Angeles; 2 Department of Obstetrics and Gynecology, University of Southern California, Los Angeles; 3 Department of Biostatistics, Mailman School of Public Health, Columbia University, New York; 4 Department of Health Sciences, California State University, Northridge; 5 Eisner Health, Los Angeles, California; 6 Sonoma Technology Inc, Petaluma, California

## Abstract

**Importance:**

Fetal growth is precisely programmed and could be interrupted by environmental exposures during specific times during pregnancy. Insights on potential sensitive windows of air pollution exposure in association with birth weight are needed.

**Objective:**

To examine the association of sensitive windows of ambient air pollution exposure with birth weight and heterogeneity by individual- and neighborhood-level stressors.

**Design, Setting, and Participants:**

Data on a cohort of low-income Hispanic women with singleton term pregnancy were collected from 2015 to 2021 in the ongoing Maternal and Developmental Risks from Environmental and Social Stressors cohort in Los Angeles, California.

**Exposures:**

Daily ambient particulate matter with aerodynamic diameter less than 10 μm (PM_10_) and aerodynamic diameter less than 2.5 μm (PM_2.5_), nitrogen dioxide (NO_2_), and 8-hour maximum ozone were assigned to residential locations. Weekly averages from 12 weeks before conception to 36 gestational weeks were calculated. Individual-level psychological stressor was measured by the Perceived Stress Scale. Neighborhood-level stressor was measured by the CalEnviroScreen 4.0.

**Main Outcomes and Measures:**

Sex-specific birth weight for gestational age *z* score (BWZ). The associations between air pollutant and BWZ were estimated using distributed lag models to identify sensitive windows of exposure, adjusting for maternal and meteorologic factors. We stratified the analyses by Perceived Stress Scale and CalEnviroScreen 4.0. We converted the effect size estimation in BWZ to grams to facilitate interpretation.

**Results:**

The study included 628 pregnant women (mean [SD] age, 22.18 [5.92] years) and their newborns (mean [SD] BWZ, −0.08 [1.03]). On average, an interquartile range (IQR) increase in PM_2.5_ exposure during 4 to 22 gestational weeks was associated with a −9.5 g (95% CI, −10.4 to −8.6 g) change in birth weight. In stratified models, PM_2.5_ from 4 to 24 gestational weeks was associated with a −34.0 g (95% CI, −35.7 to −32.4 g) change in birth weight and PM_10_ from 9 to 14 gestational weeks was associated with a −39.4 g (95% CI, −45.4 to −33.4) change in birth weight in the subgroup with high Perceived Stress Scale and high CalEnviroScreen 4.0 scores. In this same group, NO_2_ from 9 to 14 gestational weeks was associated with a −40.4 g (95% CI, −47.4 to −33.3 g) change in birth weight and, from 33 to 36 gestational weeks, a −117.6 g (95% CI, −125.3 to −83.7 g) change in birth weight. Generally, there were no significant preconception windows for any air pollutants or ozone exposure with birth weight.

**Conclusions and Relevance:**

In this cohort study, early pregnancy to midpregnancy exposures to PM_2.5_, PM_10_, and NO_2_ were associated with lower birth weight, particularly for mothers experiencing higher perceived stress and living in a neighborhood with a high level of stressors from environmental pollution.

## Key Points

**Question:**

When is the sensitive window of ambient air pollution exposure in association with birth weight?

**Findings:**

In this cohort study of 628 predominately low-income Hispanic women who were pregnant, exposures to particulate matter and nitrogen dioxide in early pregnancy to midpregnancy were significantly associated with lower birth weight, particularly for mothers experiencing higher perceived stress during the prenatal period and living in a neighborhood with a high level of stressors from environmental pollution.

**Meaning:**

The findings of this study suggest that protecting women who are pregnant from air pollution may improve birth weight, particularly among mothers with high levels of psychological stress or environmental pollution.

## Teaser

This cohort study examines the association between ambient air pollution and infant birth weight by maternal individual- and neighborhood-level stressors.

## Introduction

Newborns with low birth weight face increased risks of neonatal mortality and long-term disease risk.^1,2,3,4,5^ In addition to its genetic determinants,^6^ birth weight has been associated with prenatal environmental exposures, including ambient air pollution.^7,8,9,10,11,12,13,14,15^ Because fetal growth is precisely programmed,^16^ specific time windows in pregnancy may be particularly sensitive to the effect of environmental exposures. Such sensitive windows, if well identified, could be a compelling period of targeted intervention and provide a better understanding of environmental influences on fetal development.^17^ However, previous findings regarding sensitive windows of air pollution exposure with birth weight have been inconsistent, with each of the 3 trimesters having been identified.^18,19,20,21^ Although the whole prenatal period could be a sensitive window to air pollution exposure, erroneous estimation and inconsistencies could also be due to a temporal trend in exposure levels in adjacent time windows.^22^ Statistical approaches, such as distributed lag models (DLMs), can better handle temporal trends, thus identifying true sensitive windows.^22,23,24^ Applying DLMs is therefore needed to better assess the critical periods of air pollution exposure during pregnancy in association with birth weight.

Robust literature reports an enhanced susceptibility to the adverse effects of prenatal air pollution exposure on birth outcomes in populations with socioeconomic disadvantage and associated stressors.^25,26^ Stressors at the individual level, such as psychological perceived stress in women who are pregnant, has been assessed in few longitudinal cohort studies.^27,28^ Generally, high levels of prenatal psychosocial stress exposure have been shown to increase children’s susceptibility to asthma and neurocognitive underdevelopment in association with air pollution exposures, possibly through altered maternal allostatic load or placental function.^26,29^ Moreover, neighborhood-level stressors could arise from the total environmental pollution (eg, traffic emissions, water and soil contamination) (hereinafter, *cumulative burden*), as well as the population’s vulnerability to poor health.^30^ However, whether individual-level psychological stress and neighborhood-level cumulative burden could be associated with the susceptibility to air pollution exposure with birth weight remains unknown.

In a cohort of predominately low-income Hispanic women who were pregnant,^31^ we used DLMs to explore sensitive windows of ambient air pollution exposure in association with birth weight. Furthermore, we evaluated whether the association between air pollution and birth weight differed by individual-level psychological stress, neighborhood-level cumulative burden, or both.

## Methods

### Study Population

The Maternal and Developmental Risks from Environmental and Social Stressors (MADRES) study is an ongoing prospective pregnancy cohort study started in 2015.^31^ Briefly, MADRES participants were recruited from clinical sites that serve low-income populations in Los Angeles, California. Women were eligible if they were aged 18 years or older, within 30 weeks of gestation, singleton pregnancy, and spoke English or Spanish fluently. Exclusion criteria included HIV infection; physical, mental, or cognitive disabilities that would prevent participation; or current incarceration. Maternal written consent was obtained at the time of recruitment. Participants were compensated for their time. The institutional review board at the University of Southern California approved all aspects of this study. This analysis is reported in accordance with the Strengthening the Reporting of Observational Studies in Epidemiology (STROBE) reporting guideline.

As of September 1, 2021, 710 participants had reached delivery and had high-quality birth outcome information abstracted from electronic medical records. We excluded 12 participants who reported smoking during pregnancy and 70 participants who delivered prematurely (<37 gestational weeks). A flowchart describing the final sample size of 628 women is provided in eFigure 1 in the Supplement. Using GPower, version 3.1.9.6,^32^ we found our final sample size was sufficient to detect a greater than 4-g difference in birth weight (Cohen *f^2^*>0.01) with a power of 80% and the type I error of 0.05.

### Measurement of Ambient Air Pollution

During prenatal visits, participants completed a questionnaire on residential addresses dated from 1 year before conception through the third trimester, which was further reviewed with study staff to ensure data accuracy. Thereafter, residential addresses and mobility were prospectively collected at each follow-up visit. Geocoded daily residential address histories were assembled for each participant. Daily estimates of 24-hour average nitrogen dioxide (NO_2_), particulate matter with aerodynamic diameter less than 2.5 μm (PM_2.5_) and less than 10 μm (PM_10_), and 8-hour maximal ground-level ozone (O_3_) were assigned to each participant’s residential location from 12 weeks before conception to 36 weeks 7 days during pregnancy, using inverse-distance-squared weighted spatial interpolation from ambient air quality monitoring data (US Environmental Protection Agency Air Quality System), with an average of 4 monitoring stations within 8 to 14 km to each residential address (eTable 2 in the Supplement). The 12 weeks before conception were chosen as a plausible biologically relevant window of exposure that may affect ovulation and the maternal and intrauterine environment close to conception. To reduce impacts from daily fluctuations, we calculated weekly average levels of each pollutant concentration.

### Measurement of Birth Weight

Birth weight and newborn sex were abstracted from electronic medical records. Gestational age at birth (median, 39.4 weeks; range, 37.0-42.4 weeks) was estimated based on ultrasonography measurement (87.6%), medical consensus (11.5%), or last menstrual period (0.9%). We calculated sex-specific birth weight for gestational age *z* scores (BWZ).^33^

### Covariates

We a priori selected the following modifiers: perceived stress during pregnancy measured by the Perceived Stress Scale (PSS)^34^ to represent individual-level psychological stressor, and the cumulative impact score from the California statewide environmental justice screening tool, CalEnviroScreen 4.0 (CES), to represent the neighborhood-level cumulative burden from both total environmental pollution and population vulnerability.^30^ The PSS is a widely used, validated 10-item psychological instrument to assess stressors that may possibly influence mental health of the respondent (how the stressor is perceived),^34^ which may be more relevant to a list of stressors that will affect one’s mental health. All available measurements of PSS scores (mean [SD], 2.24 [0.89] measurements per participant) in the first (mean PSS, 12.8 [6.4]), second (12.2 [6.0]), or third (11.2 [5.8]) trimesters were averaged as the final PSS score across pregnancy. We chose the upper quartile (75th percentile) to dichotomize a high-PSS group (≥16 PSS score) and a low-PSS group (<16). The CalEnviroScreen 4.0 was developed by the California Office of Environmental Health Hazard Assessment to identify census tracts in California that are disproportionately burdened by both multiple sources of pollution (eg, air, water, and soil) and high population vulnerability (eg, a higher proportion of poverty, low educational level, prevalence of asthma, and low birth weight).^30^ The CES cumulative impact score ranges from 0 to 100, with a higher score indicating a higher cumulative burden, and the score was assigned to each participant based on residential census tract.^30^ We chose the California state median to dichotomize a high CES group (≥50) and a low CES group (<50).

Potential confounders were selected a priori based on literature review and analyses (eFigure 2 in the Supplement).^35^ Participants self-reported prepregnancy weight, race, Hispanic ethnicity, birth country, and marital status. The enrollment time point was determined based on the gestational week of consent (<20 vs 20-30 weeks). Maternal standing height was measured twice by a stadiometer. Maternal prepregnancy body mass index was calculated using self-reported prepregnancy weight and measured height at a prenatal study visit. We created a combined variable indicating ethnicity by birthplace, which had 3 categories: non-Hispanic, Hispanic born in the US, and Hispanic born outside the US. Marital status was categorized to indicate cohabitation status, including cohabitate, not cohabitate, and decline to respond. Birth order was combined as first, second, and third or more. To maximize statistical power, we coded missing observations of categorical covariates as a separate category. Weekly outdoor temperature was calculated using daily average temperatures at the residential location extracted from a high-resolution (4 × 4 km) gridded surface meteorologic data set.^36^

### Statistical Analysis

We examined frequencies and proportions to describe the distributions of categorical variables. Birth weight for gestational age *z* score was described by mean (SD) and tested for differences by covariates using analysis of variance. We used DLMs to examine the association of weekly exposure to each of the air pollutants (ie, PM_2.5_, PM_10_, NO_2_, and O_3_) with BWZ.^23^ The DLMs simultaneously included exposure to each air pollutant’s weekly levels from 12 weeks before conception to 36 gestational weeks with a cross-basis, which combined a linear dose-response function and a nonlinear lag-response function with a 3-knot natural cubic spline to constrain correlated weekly exposures.^23^ The number and location of knots for each air pollutant were chosen based on the Akaike information criterion and visual inspections.^23^ Distributed lag models were adjusted for the aforementioned potential confounders and weekly temperature. Effect size estimates are presented as changes in BWZ per each IQR increase in air pollutant (ie, 4 μg/m^3^ for PM_2.5,_12 μg/m^3^ for PM_10,_ 11 ppb for NO_2_, and 15 ppb for O_3_). To facilitate interpretation, we converted the difference in BWZ to the difference in birth weight (grams), with 1 *z* score = 450 g (the SD of birth weight at the 40th gestational week in our sample). We used the bayesian distributed lag interaction models and identified that a heterogeneity of both a sensitive window and within-window estimate fitted our data best.^37^ Therefore, we stratified the analyses by PSS (high vs low), CES (high vs low), and their joint distribution. We conducted sensitivity analyses, including multipollutant DLMs with all air pollutants included as separate cross-bases, complete-case analysis by removing participants with missing values of covariates, DLMs with different number and location of knots in the cross-basis, and DLMs adjusting for PSS, CES, or both as covariates. A *P* value <.05 was deemed statistically significant. Analyses were conducted with in R, 4.2.0 with package dlnm, version 2.4.7 and regime (R Foundation for Statistical Analysis); all others were performed with SAS, version 9.4 (SAS Institute LLC). Further details are presented iMen the eMethods and eTable 1 in the Supplement.

## Results

The study included 628 pregnant women and their newborns (mean [SD] BWZ, −0.08 [1.03]). The Table presents population characteristics and mean (SD) of BWZ by categories of each characteristic. Participants generally were from lower socioeconomic status households, with 256 (40.8%) reporting an annual household income less than $30 000, and 327 (52.1%) had high school or lower educational level. A total of 396 of 626 participants with available data (63.3%) of participants lived in neighborhoods with a CES cumulative impact score above the California median, indicating a high proportion of participants from neighborhoods with a high cumulative burden. Among 543 participants with at least 1 completed PSS questionnaire, 132 (24.3%) had a PSS score greater than or equal to 16 (upper quartile). The BWZ was lower in the high-PSS group compared with the low-PSS group (mean difference, −0.16; 95% CI, −0.36 to 0.04) and was comparable between low- and high-CES groups (mean difference, −0.06; 95% CI, −0.22 to 0.11). The distribution of ambient air pollutant concentrations is provided in eTable 3 in the Supplement. Distribution of covariates, BWZ, and air pollution by the subgroups of PSS and CES is provided in eTable 4 in the Supplement.

**Table.** Population Characteristics and BWZ Among 628 Participants in the MADRES Cohort

Characteristic	No. (%)	BWZ, mean (SD)	*P* value
Overall	628 (100)	−0.08 (1.03)	
Enrollment time point			
Regular entry (<20 wk)	461 (73.4)	−0.01 (1.03)	.006
Late entry (20-30 wk)	167 (26.6)	−0.26 (2.98)	
Language preference			
English	423 (67.4)	−0.10 (1.33)	.41
Spanish	203 (32.3)	−0.03 (1.11)	
Missing	2 (0.3)	0.78 (2.29)	
Maternal country of origin			
Latin America^a^	225 (35.8)	0.06 (1.12)	.01
US	280 (44.6)	−0.09 (7.98)	
Other/unknown^b^	123 (19.6)	−0.28 (1.12)	
Maternal ethnicity			
Hispanic	463 (73.7)	0.01 (1.09)	.001
Non-Hispanic	130 (20.7)	−0.29 (1.15)	
Missing	35 (5.6)	−0.44 (1.12)	
Cohabitation status			
Cohabitate with spouse or partner	373 (59.4)	−0.06 (1.22)	.74
Noncohabitate	131 (20.9)	−0.05 (1.84)	
Missing/decline to respond	124 (19.7)	−0.14 (1.15)	
Annual household income, $			
<15 000	110 (17.5)	0.00 (1.58)	.07
15 000-29 000	146 (23.2)	−0.00 (5.92)	
≥30 000	138 (22.0)	−0.00 (1.84)	
Unknown	200 (31.8)	−0.15 (1.04)	
Missing	34 (5.4)	−0.49 (1.89)	
Educational level			
Below 12th grade	150 (23.9)	0.03 (1.33)	.10
Completed 12th grade	177 (28.2)	−0.05 (5.97)	
Some college	164 (26.1)	−0.09 (1.14)	
College or above	103 (16.4)	−0.11 (7.99)	
Missing	34 (5.4)	−0.49 (1.89)	
Prepregnancy BMI category			
Normal/underweight	204 (32.5)	−0.33 (1.97)	<.001
Overweight	194 (30.9)	−0.06 (1.99)	
Obese	227 (36.1)	0.14 (1.73)	
Missing	3 (0.5)	−0.17 (1.06)	
Perceived Stress Scale scores			
Low (<16)	411 (65.4)	−0.02 (1.01)	.13
High (≥16)	132 (21.0)	−0.17 (1.05)	
Missing	85 (13.5)	−0.20 (1.05)	
CalEnviroScreen 4.0 Cumulative Impact score			
Low (<50)	230 (36.6)	−0.11 (1.08)	.51
High (≥50)	396 (63.1)	−0.06 (0.99)	
Missing	2 (0.3)	−0.12 (0.14)	
Birth order			
First born	191 (30.4)	−0.27 (1.52)	.001
Second born	167 (26.6)	−0.03 (6.99)	
Third or later born	167 (26.6)	0.16 (1.37)	
Missing	103 (16.4)	−0.15 (1.11)	
Newborn sex			
Female	322 (51.3)	−0.00 (1.97)	.09
Male	306 (48.7)	−0.14 (9.98)	

Abbreviations: BMI, body mass index; BWZ, birth weight for gestational age *z* score; MADRES, Maternal and Developmental Risks from Environmental and Social Stressors.

^a^ This category includes small numbers of participants from Chile, Colombia, El Salvador, Guatemala, Honduras, Mexico, Nicaragua, and Venezuela.

^b^ N = Twenty-two for other country of origin, 101 for unknown owing to missing data.

**Figure 1.**
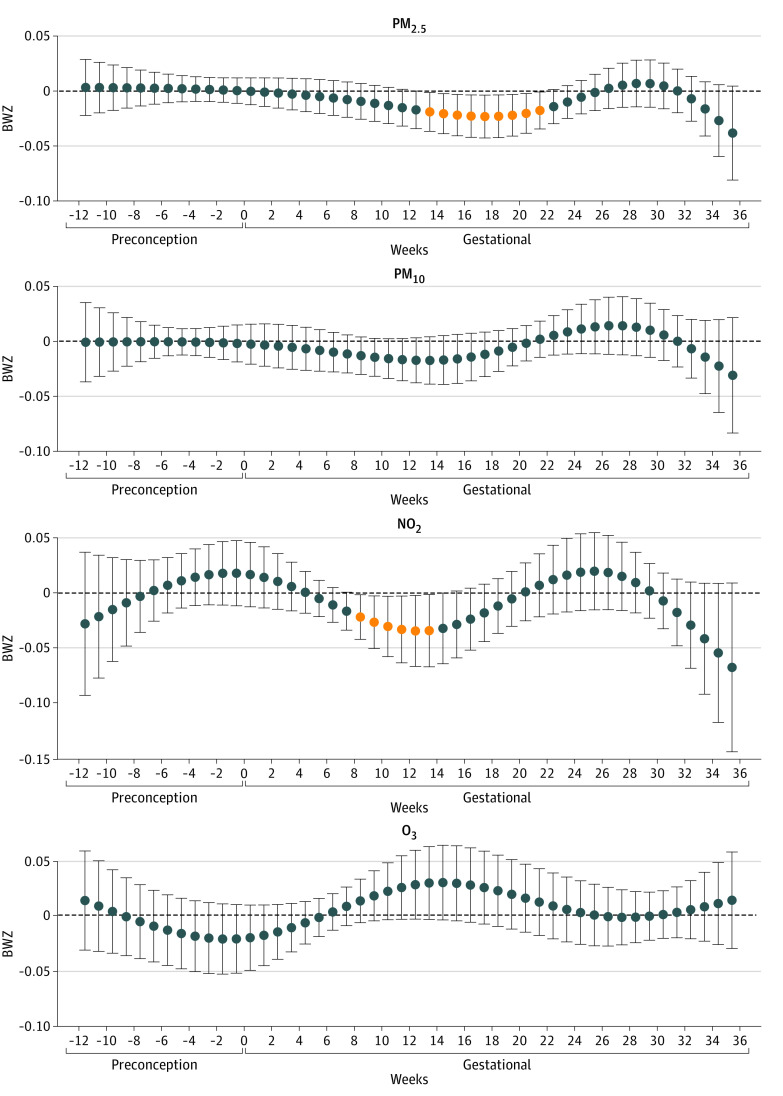
Associations of Weekly Air Pollution Exposure With Gestational Age *z* Score (BWZ) Among 628 Newborns in the Maternal and Developmental Risks from Environmental and Social Stressors Cohort All results were from distributed lag models adjusted for weekly temperature, maternal age, educational level, maternal prepregnancy body mass index, maternal ethnicity and nativity, cohabitation status, birth order, and enrollment time. Outcome estimation was based on per IQR increases in each air pollutant: particulate matter with aerodynamic diameter less than 2.5 μm (PM_2.5_), 4 μg/m^3^; particulate matter with aerodynamic diameter less than 10 μm (PM_10_), 12 μg/m^3^; nitrogen dioxide (NO_2_), 11 ppb; and ozone (O_3_), 15 ppb. Error bars indicate 95% CI.

Figure 1 presents the adjusted association of weekly exposure to each air pollutant with BWZ in the overall population. The mean difference in BWZ and birth weight by each IQR increase in air pollutants during each identified sensitive window can be found in eTable 5 in the Supplement. The IQR increase in PM_2.5_ exposure during the window from 14 to 22 gestational weeks was associated with lower birth weight. On average, birth weight changed by −9.5 g (95% CI, −10.4 to −8.6 g) per IQR increase in PM_2.5_ exposure in each week across this sensitive window, with the largest change (−10.4 g; 95% CI, −19.2 to −1.6 g) in gestational week 18. An IQR increase in NO_2_ exposure in each week from 9 to 14 gestational weeks was associated with birth weight (−13.5 g; 95% CI, −15.6 to −11.5 g). There was no association of PM_10_ and O_3_ exposure with birth weight. The results were robust in sensitivity analyses of multipollutants (eFigure 3 in the Supplement), adjusting for CES or PSS (eFigure 4 in the Supplement), various placement of cross-basis knots (eFigure 5 in the Supplement), or complete case analysis (eFigure 6 in the Supplement).

**Figure 2.**
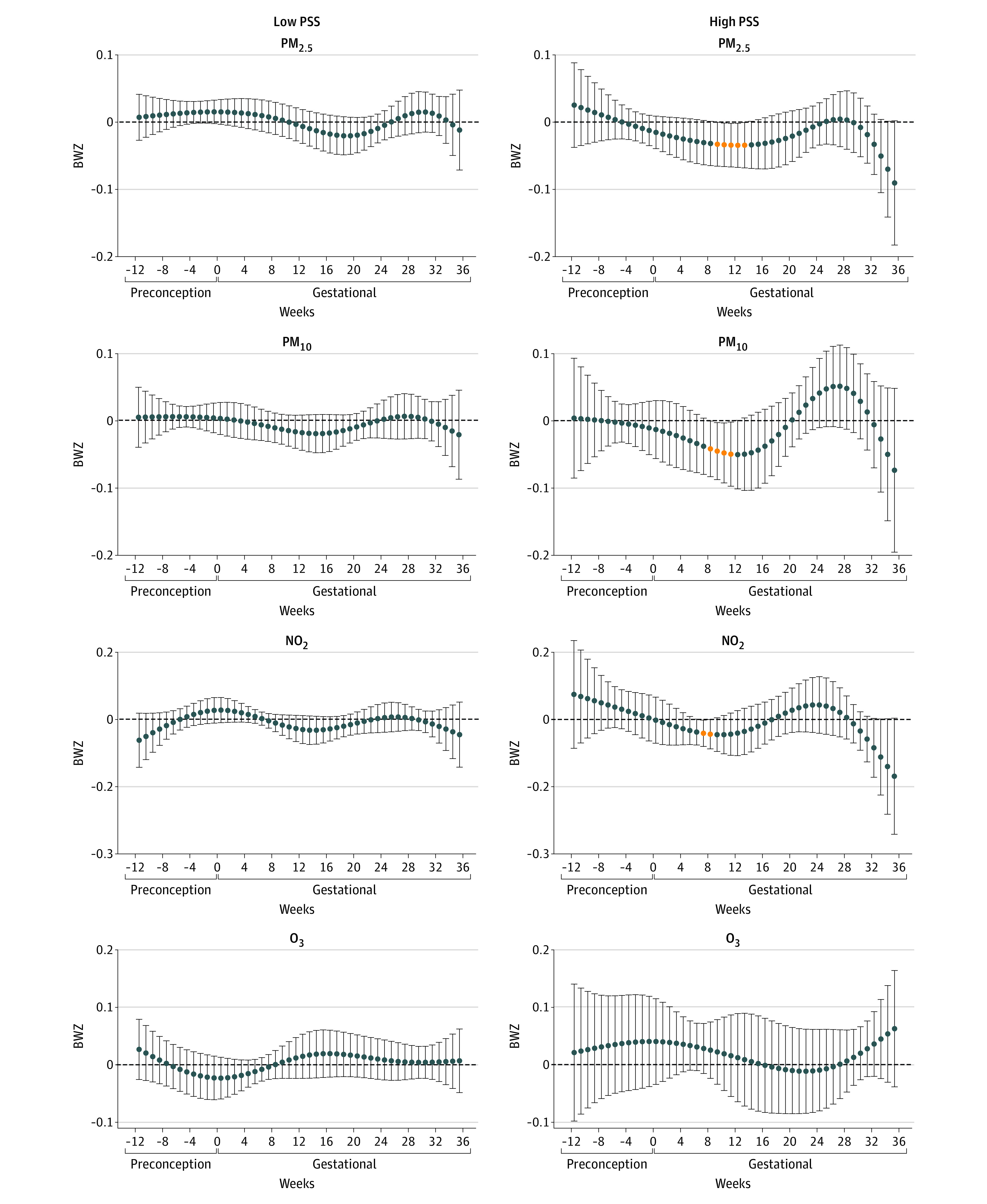
Stratification Analysis by Individual-Level Stressor Perceived Stress Scale (PSS) Associations among 411 participants with low PSS and associations among 132 participants with high PSS scores. Associations were adjusted for weekly temperature, maternal age, educational level, prepregnancy body mass index, ethnicity and nativity, educational level, cohabitation status, birth order, and enrollment time point. Outcome estimation was based on per IQR increases in each air pollutant: particulate matter with aerodynamic diameter less than 2.5 μm (PM_2.5_), 4 μg/m^3^; particulate matter with aerodynamic diameter less than 10 μm (PM_10_), 12 μg/m^3^; nitrogen dioxide (NO_2_), 11 ppb; and ozone (O_3_), 15 ppb.

**Figure 3.**
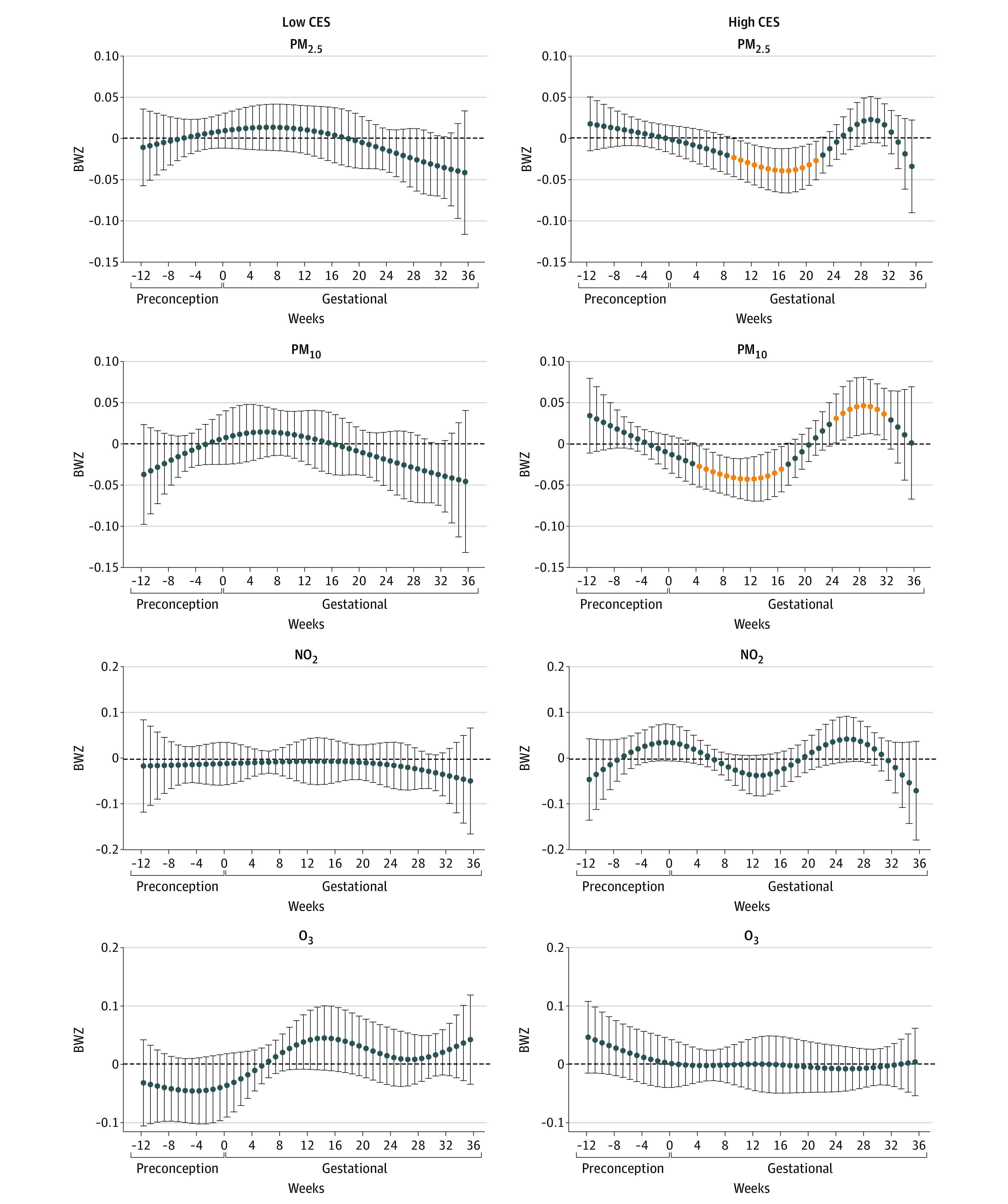
Stratification Analysis by Neighborhood-Level Stressor CalEnviroScreen 4.0 (CES) A, Associations among 230 participants with low CES. B, Associations among 396 participants with high CES. Associations were adjusted for weekly temperature, maternal age, educational level, prepregnancy body mass index, ethnicity and nativity, marital status, and birth order. Outcome estimation was based on per IQR increases in each air pollutant: particulate matter with aerodynamic diameter less than 2.5 μm (PM_2.5_), 4 μg/m^3^; particulate matter with aerodynamic diameter less than 10 μm (PM_10_), 12 μg/m^3^; nitrogen dioxide (NO_2_), 11 ppb; and ozone (O_3_), 15 ppb.

**Figure 4.**
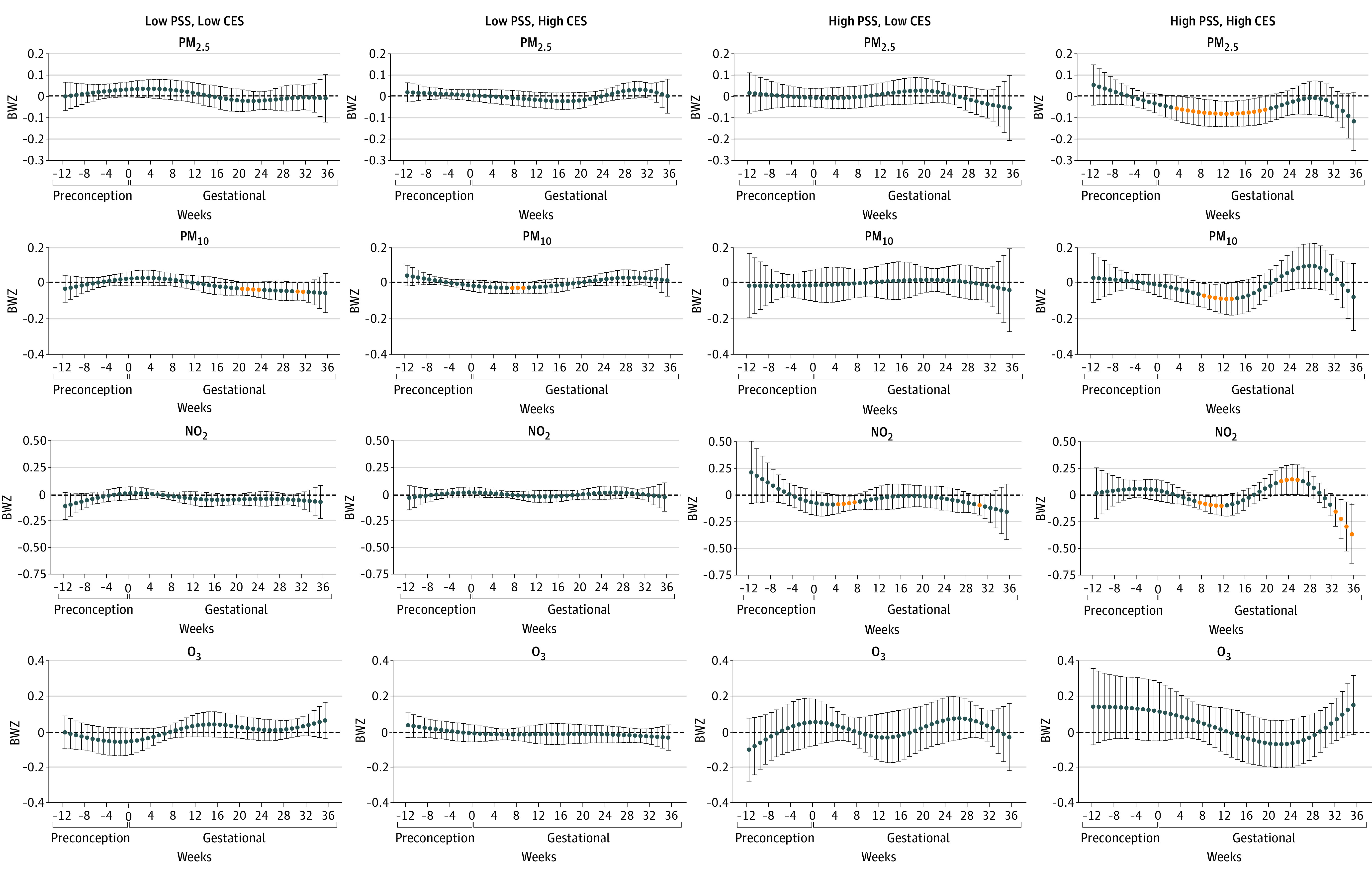
Stratification Analysis by Combined Groups of Perceived Stress Scale (PSS) and CalEnviroScreen 4.0 (CES) Sample sizes in subgroups were 150 for low PSS and CES scores, 260 for low PSS scores and high CES scores, 49 for high PSS scores and low CES scores, and 82 for high PSS and CES scores. Associations were adjusted for weekly temperature, maternal age, educational level, prepregnancy body mass index, ethnicity and nativity, marital status, and birth order. Effect size estimation was based on per IQR increases in each air pollutant: particulate matter with aerodynamic diameter less than 2.5 μm (PM_2.5_), 4 μg/m^3^; particulate matter with aerodynamic diameter less than 10 μm (PM_10_), 12 μg/m^3^; nitrogen dioxide (NO_2_), 11 ppb; and ozone (O_3_), 15 ppb.

In stratification analysis by PSS (Figure 2), we observed inverse associations of PM_2.5_, PM_10_, and NO_2_ with birth weight in the high-PSS group (n = 132), but no associations in the low-PSS group (n = 411). In stratification analysis by CES (Figure 3), we observed associations of PM_2.5_ and PM_10_ with birth weight in the high-CES group (n = 396), but no associations in the low-CES group (n = 230). We further stratified the analysis by the combined groups of PSS and CES scores (Figure 4). In the high-PSS, high-CES group (n = 82), we found wide sensitive windows of PM_2.5_ and PM_10_ exposure with inverse associations with birth weight: an IQR increase in PM_2.5_ exposure in each week from 4 to 20 gestational weeks (−34.0 g; 95% CI, −35.7 to −32.4 g) and in PM_10_ exposure in each week from 9 to 14 gestational weeks (−39.4 g; 95% CI, −45.4 to −33.4 g). Also in this group, an IQR increase in NO_2_ exposure was associated with birth weight in each week from 9 to 14 weeks (−40.4 g; 95% CI, −47.4 to −33.3 g), and from 33 to 36 weeks (−117.6 g; 95% CI, −125.3 to −83.7 g) in birth weight. An IQR increase in NO_2_ exposure in each week from 23 to 26 weeks also was associated with birth weight (63.2 g; 95% CI, 47.8 to 78.5 g). Some sparse significant differences in BWZ by PM_10_ in the low-PSS, high-CES group and by NO_2_ in the high-PSS, low-CES group were also seen, both within the window from 5 to 10 gestational weeks. There were no associations between O_3_ and birth weight.

## Discussion

In the predominately low-income Hispanic population that comprises the MADRES cohort, we observed robust associations of lower birth weight with NO_2_, PM_2.5_, and PM_10_ exposures in the window from early pregnancy to midpregnancy. The inverse associations were generally stronger, and the sensitive windows were generally wider within subgroups with either high individual-level perceived stress, high neighborhood-level cumulative burden, or both. We found no evidence for associations of air pollution exposure during the preconception period with birth weight nor for prenatal O_3_ exposure with birth weight.

A robust literature exists reporting the inverse association of ambient PM_2.5_ and PM_10_ exposure with birth weight, and our results are consistent with these overall findings. In a meta-analysis of 17 studies, increases in PM_2.5_ exposure during the entire pregnancy were associated with lower birth weight with an effect size similar to ours.^38^ We found exposure to PM_10_ from 5 to 17 gestational weeks was associated with lower birth weight in women who had high perceived stress, lived in a neighborhood with a high cumulative burden, or both, but not in the overall cohort, suggesting outcomes of PM_10_ may be sensitive to population characteristics that have also been noted in previous studies.^10,20^ Other studies, including a meta-analysis, reported inconsistent associations of NO_2_ with birth weight.^14,39^ We found an association of NO_2_ exposure during the sensitive window from 9 to 14 gestational weeks with lower birth weight in the overall cohort that was strengthened among the subgroup with high perceived stress but attenuated toward the null in the low perceived stress subgroup. These findings together suggest a critical role for stressors at individual and neighborhood levels in determining the associations between sensitive windows of various air pollutants and birth weight.

Previous studies aiming to identify associations between sensitive windows of PM_2.5_ exposure and birth weight have identified inconsistent sensitive windows ranging from early to late pregnancy.^9,10,11^ In the present study, our identification of early pregnancy to midpregnancy as a sensitive window is consistent with other studies.^8,9,10,14,15^ Registry-based studies based in China^12,13^ and Texas^21^ have also used DLMs to identify sensitive windows in finer periods (eg, by week or month), and generally have found the exposure window of 20 to 35 gestational weeks to be important, which partly overlaps with our findings. Early pregnancy to midpregnancy is the critical period of organogenesis and functional initiation that lays the foundations for fetal growth, such as the beginning of hematopoiesis, brown fat formation, and thyroid hormone secretion.^16^ Therefore, exposure to air pollution during early pregnancy could affect fetal and placental development and eventually lead to lower birth weight, particularly for mothers with high psychosocial stress.

Both individual-level psychosocial stress and neighborhood-level cumulative burden could amplify the inverse association of PM_2.5_, PM_10_, and NO_2_ exposure with birth weight. Our findings are consistent with studies that found neighborhood-level socioeconomic deprivation is associated with prenatal exposure to air pollution and offspring health outcomes, including birth weight.^25,26,40,41^ Among a handful of studies that have measured individual-level psychological stress, susceptibility to asthma, poor lung function, or accelerated biological aging associated with air pollution exposures was greater in a higher stress group than in the lower stress group.^27,28^ To our knowledge, our study is the first to report that individual-level maternal perceived stress may increase susceptibility to changes in birth weight associated with exposure to PM_2.5_, PM_10_, and NO_2_ exposure and widens the sensitive windows of exposure.

The CES cumulative impact scores capture the neighborhood-level cumulative burden from both environmental pollution and population vulnerability, thus providing further evidence that susceptibility to changes in birth weight associated with air pollution was greater among populations with a high cumulative neighborhood burden. The population with both high individual-level psychosocial stress and high neighborhood-level cumulative burden faced the greatest susceptibility and the widest sensitive window of air pollution exposure in association with birth weight, suggesting that individual-level and neighborhood-level stressors may compound one another. Both individual- and neighborhood-level stressors might act on some known biological pathways that are also involved in responses to air pollution exposures, including heightened oxidative stress and systemic inflammation, hormonal interruption, and reduced placental function.^42,43,44,45^ Nevertheless, further studies are needed to elucidate the mechanisms of the joint effects of individual and neighborhood stressors on the association between air pollution and reduced fetal growth.

### Strengths and Limitations

A strength of our study is the use of DLMs to identify sensitive windows of exposure and the use of individual- and neighborhood-level stressors to investigate outcome modification.

The study has limitations, including lack of information on the source and constituents of particulate matter. Neighborhood-burden CES cumulative impact score is only available in California, but similar measures, such as the Environmental Justice Screening, are available for all US states.^46^ Sample size was reduced in subgroup analyses, especially in the case of PSS missingness (13.5%). We excluded preterm births owing to the missingness of exposure before 37 weeks, which could potentially introduce collider bias. We attempted to evaluate such bias by comparing exposure and covariates between preterm and full-term infants (eTable 5 in the Supplement) and by controlling for known covariates associated with both preterm status and birth weight. Although we cannot rule out the possibility of collider bias, we believe such bias would be nonsignificant. Our analyses involve many statistical tests; thus, some of the associations, especially those not consistently seen in subgroup analyses, might be identified by chance.

## Conclusions

In this cohort study, ambient PM_2.5_, PM_10_, and NO_2_ during sensitive windows primarily in early pregnancy to midpregnancy were associated with lower birth weight. Wider sensitive windows and greater changes were seen in children born to mothers with high perceived stress or who lived in a health-disparity neighborhood with a high burden from environmental pollution and population vulnerability.

## Notes

Supplement. **eMethods.** Detailed Methods **eTable 1.** BDLIM Model Fitting of Various Effect Heterogeneity by the Joint Distribution of PSS and CES **eTable 2.** Number of Close-by Air Quality Monitoring Stations and Distance to Nearest Air Quality Monitoring Station in the Overall Population and by CES **eTable 3.** Distribution of Air Pollutant Concentrations and Temperature Among 628 Participants in the MADRES Cohort **eTable 4A.** Population Characteristics and Birth Weight z Scores (BWZ) According to Individual and Neighborhood Level Stressors **eTable 4B.** Population Characteristics and Birth Weight *z* Scores (BWZ) According to the Joint Distribution of Individual and Neighborhood Level Stressors **eTable 5.** Average Weekly Effect Estimation of Air Pollution Exposure in Identified Sensitive Windows on Birth Weight **eTable 6.** Population Characteristics and Birth Weight *z* Scores (BWZ) According to Preterm Birth Status Among All Participants in the MADRES Cohort (2015-2021) **eFigure 1.** Flow Chart of Participants Included in This Analysis From the MADRES Cohort **eFigure 2.** Theoretical Causal Structure Underlying the Association of Air Pollution Exposure With BWZ **eFigure 3.** Sensitivity Analysis With Multiple Pollutants in DLM **eFigure 4.** Sensitivity Analysis Adjusting for CES, PSS, or Both **eFigure 5A.** Sensitivity Analysis With Various Knots Placement for PM_2.5_ and PM_10_ **eFigure 5B.** Sensitivity Analysis With Various Knots Placement for NO_2_ and O_3_ **eFigure 6.** Sensitivity Analysis With Complete Cases
