# Numeric Error in Abstract and Omission in Results

**DOI:** 10.1001/jamanetworkopen.2022.45389

**Published:** 2022-11-15

In the Original Investigation titled “Association Between Ambient Air Pollution and Birth Weight by Maternal Individual- and Neighborhood-Level Stressors,”^1^ published October 25, 2022, there was a numeric error in the Abstract Results. The mean age given as 22.18 should have been 28.18. In addition, the mean (SD) age for the population of pregnant women was not reported in the Results. The parenthetical phrase “(mean [SD] age, 28.18 [5.92] years)” was added to the Results. This article has been corrected.^1^
