# Supplementary material for: Association Between Ambient Air Pollution and Birth Weight by Maternal Individual- and Neighborhood-Level Stressors
Source: JAMA Netw Open. 2022 Oct 25;5(10):e2238174. doi: 10.1001/jamanetworkopen.2022.38174 (PMC9597392; doi:10.1001/jamanetworkopen.2022.38174)
Supplement: Supplement. — eMethods. Detailed Methods eTable 1. BDLIM Model Fitting of Various Effect Heterogeneity by the Joint Distribution of PSS and CES eTable 2. Number of Close-by Air Quality Monitoring Stations and Distance to Nearest Air Quality Monitoring Station in the Overall Population and by CES eTable 3. Distribution of Air Pollutant Concentrations and Temperature Among 628 Participants in the MADRES Cohort eTable 4A. Population Characteristics and Birth Weight z Scores (BWZ) According to Individual and Neighborhood Level Stressors eTable 4B. Population Characteristics and Birth Weight z Scores (BWZ) According to the Joint Distribution of Individual and Neighborhood Level Stressors eTable 5. Average Weekly Effect Estimation of Air Pollution Exposure in Identified Sensitive Windows on Birth Weight eTable 6. Population Characteristics and Birth Weight z Scores (BWZ) According to Preterm Birth Status Among All Participants in the MADRES Cohort (2015-2021) eFigure 1. Flow Chart of Participants Included in This Analysis From the MADRES Cohort eFigure 2. Theoretical Causal Structure Underlying the Association of Air Pollution Exposure With BWZ eFigure 3. Sensitivity Analysis With Multiple Pollutants in DLM eFigure 4. Sensitivity Analysis Adjusting for CES, PSS, or Both eFigure 5A. Sensitivity Analysis With Various Knots Placement for PM2.5 and PM10 eFigure 5B. Sensitivity Analysis With Various Knots Placement for NO2 and O3 eFigure 6. Sensitivity Analysis With Complete Cases [file jamanetwopen-e2238174-s001.pdf]

Supplementary Online Content

Niu Z, Habre R, Chavez TA, et al. Association between ambient air pollution and birth weight by maternal individual- and neighborhood-level stressors. *JAMA Netw Open*. 2022;5(10):e2238174. doi:10.1001/jamanetworkopen.2022.38174

eMethods. Detailed Methods

eTable 1. BDLIM Model Fitting of Various Effect Heterogeneity by the Joint Distribution of PSS and CES

eTable 2. Number of Close-by Air Quality Monitoring Stations and Distance to Nearest Air Quality Monitoring Station in the Overall Population and by CES

eTable 3. Distribution of Air Pollutant Concentrations and Temperature Among 628 Participants in the MADRES Cohort

eTable 4A. Population Characteristics and Birth Weight z Scores (BWZ) According to Individual and Neighborhood Level Stressors

eTable 4B. Population Characteristics and Birth Weight *z* Scores (BWZ) According to the Joint Distribution of Individual and Neighborhood Level Stressors

eTable 5. Average Weekly Effect Estimation of Air Pollution Exposure in Identified Sensitive Windows on Birth Weight

eTable 6. Population Characteristics and Birth Weight *z* Scores (BWZ) According to Preterm Birth Status Among All Participants in the MADRES Cohort (2015-2021)

eFigure 1. Flow Chart of Participants Included in This Analysis From the MADRES Cohort

eFigure 2. Theoretical Causal Structure Underlying the Association of Air Pollution Exposure With BWZ

eFigure 3. Sensitivity Analysis With Multiple Pollutants in DLM

eFigure 4. Sensitivity Analysis Adjusting for CES, PSS, or Both

eFigure 5A. Sensitivity Analysis With Various Knots Placement for $PM_{2.5}$ and PM_{10}
eFigure 5B. Sensitivity Analysis With Various Knots Placement for NO_2 and O_3

eFigure 6. Sensitivity Analysis With Complete Cases

This supplementary material has been provided by the authors to give readers additional information about their work.

© 2022 Niu Z et al. *JAMA Network Open.*

eMethods. Detailed Methods

All DLM included weekly temperature modeled with a cross-basis function of a 2-*df* natural cubic spline for the dose-response function and a 3-*df* a natural cubic spline for the lag-response function We additionally applied the Bayesian Distributed Lag Interaction Models (BDLIM) to our data using the joint distribution of PSS and CES as the effect modifier. This analysis enabled us to determine how our data fit best for the heterogeneity among four possible patterns: a heterogeneity of the sensitive window only (BDLIM-w), a heterogeneity of the within-window effect only (BDLIM-b), a heterogeneity of both a sensitive window and within-window effect (BDLIM-bw), or no heterogeneity (BDLIM-n). BDLIM-n indicates no heterogeneity by the effect modifier, thus a homogeneous DLM fits the data best1. Similarly, BDLIM-bw supports that both the sensitive window and the effect within the sensitive window are different by the effect modifier, thus a stratified DLM fits the data best. Detailed information on the methodological development of BDLIM can be found from in the paper by Wilson et al.[1] As Wilson et al suggested, the best model fit can be determined by comparing the mean log posterior predictive density (MLPPD), where a larger MLPPD indicating a higher probability of effect heterogeneity pattern identification. As shown in the table below, analyses with $PM_{2.5}$, PM_{10}, NO_2 all support that BDLIM-bw has the highest probability (84.4%, 46.05, 98.7% for $PM_{2.5}$, PM_{10}, NO_2, respectively) of effect heterogeneity pattern. BDLIM-w has the highest MLPPD for the analyses with O_3, but there was no meaningful association between weekly O_3 exposure with birthweight in the first place. Therefore, results from the BDLIM analysis supported our use of stratified DLM. DLM/BDLIM analyses were conducted in R (4.2.0) with package dlnm (v2.4.7) and regime, all others in SAS (v9.4).

© 2022 Niu Z et al. *JAMA Network Open.*

eTable 1. BDLIM Model fitting of various effect heterogeneity by the joint distribution of PSS and CES

Air Pollutant	Model	DIC	pD	MLPPD
$PM_{2.5}$	BDLIM_bw	1566.628	37.872	0.844
	BDLIM_w	1554.055	23.406	0.127
	BDLIM_b	1560.14	28.014	0.029
	BDLIM_n	1561.694	23.266	0
PM_{10}	BDLIM_bw	1569.199	36.485	0.46
	BDLIM_b	1556.694	23.707	0.35
	BDLIM_w	1554.318	20.719	0.19
	BDLIM_n	1562.933	23.949	0.001
NO_2	BDLIM_bw	1566.331	37.743	0.987
	BDLIM_b	1558.76	25.737	0.012
	BDLIM_w	1556.679	21.187	0.001
	BDLIM_n	1559.681	23.055	0
O_3	BDLIM_w	1558.259	22.596	0.511
	BDLIM_b	1561.528	25.753	0.456
	BDLIM_bw	1575.655	37.22	0.032
	BDLIM_n	1564.636	22.632	0.001

Note: DIC – deviance information criterion; pD – predictive density; MLPPD – mean log posterior predictive density.

eReference

1. Wilson A, Chiu YM, Hsu HL, Wright RO, Wright RJ, Coull BA. Bayesian distributed lag interaction models to identify perinatal windows of vulnerability in children's health. Biostatistics (Oxford, England). 2017;18(3):537-552.

© 2022 Niu Z et al. *JAMA Network Open.*

eTable 2. Number of close-by air quality monitoring stations and distance to nearest air quality monitoring station in the overall population and by CES

	Mean (SD)			
	**Overall sample**	**CES<50**	**CES≥50**	**P-value**
**Number of close-by air quality monitoring station**				
NO_2 (ppb)	3.97 (0.26)	3.94 (0.38)	3.98 (0.15)	0.08
O_3 (ppb)	3.97 (0.20)	3.95 (0.29)	3.99 (0.12)	0.06
$PM_{2.5}$ (ug/m^3)	3.98 (0.19)	3.96 (0.28)	3.99 (0.10)	0.09
PM_{10} (ug/m3)	3.80 (0.41)	3.75 (0.51)	3.83 (0.34)	0.03
**Distance to nearest air quality monitoring station (km)**				
NO_2 (ppb)	7.52 (2.60)	7.82 (3.04)	7.35 (2.29)	0.05
O_3 (ppb)	7.66 (2.53)	7.98 (3.03)	7.47 (2.18)	0.03
$PM_{2.5}$ (ug/m^3)	8.49 (3.34)	9.19 (3.91)	8.09 (2.89)	<0.001
PM_{10} (ug/m3)	14.58 (8.21)	14.69 (8.39)	14.51 (8.11)	0.79

© 2022 Niu Z et al. *JAMA Network Open.*

eTable 3. Distribution of air pollutant concentrations and temperature among 628 participants in the MADRES cohort

Period	Air Pollutant	Mean (SD)	Median (Q1, Q3)
Whole pregnancy	NO_2 (ppb)	16.70 (2.92)	16.78 (14.65 - 18.74)
	O_3 (ppb)	42.44 (3.77)	41.55 (39.99 - 44.31)
	$PM_{2.5}$ (ug/m^3)	12.05 (1.12)	12.03 (11.24 - 12.94)
	PM_{10} (ug/m^3)	29.13 (4.76)	29.73 (25.42 - 33.04)
	Temperature (°C)	19.25 (1.33)	19.33 (18.44 - 20.11)
12 weeks before conception	NO_2 (ppb)	17.41 (5.86)	17.39 (12.51 - 21.57)
	O_3 (ppb)	41.86 (7.53)	43.44 (35.49 - 47.43)
	$PM_{2.5}$ (ug/m^3)	11.91 (2.21)	11.67 (10.50 - 12.97)
	PM_{10} (ug/m^3)	29.43 (5.76)	29.25 (24.80 - 33.25)
	Temperature (°C)	18.93 (3.27)	18.61 (16.17 - 21.82)
1st trimester	NO_2 (ppb)	16.90 (6.05)	15.53 (11.95 - 21.70)
	O_3 (ppb)	42.37 (7.10)	44.31 (36.88 - 47.13)
	$PM_{2.5}$ (ug/m^3)	11.93 (2.30)	11.50 (10.50 - 13.11)
	PM_{10} (ug/m^3)	29.41 (5.94)	29.47 (24.68 - 33.01)
	Temperature (°C)	19.02 (3.33)	18.84 (16.49 - 21.85)
2nd trimester	NO_2 (ppb)	16.44 (5.79)	15.34 (11.71 - 20.83)
	O_3 (ppb)	42.71 (7.05)	44.35 (36.86 - 47.06)
	$PM_{2.5}$ (ug/m^3)	12.07 (2.20)	11.94 (10.71 - 13.27)
	PM_{10} (ug/m^3)	29.18 (5.90)	29.63 (24.36 - 33.27)
	Temperature (°C)	19.40 (3.21)	19.28 (16.59 - 22.33)
3rd trimester	NO_2 (ppb)	16.78 (5.92)	15.74 (11.73 - 21.50)
	O_3 (ppb)	42.15 (7.80)	43.35 (36.06 - 47.30)
	$PM_{2.5}$ (ug/m^3)	12.15 (2.32)	12.04 (10.61 - 13.40)
	PM_{10} (ug/m^3)	28.71 (6.42)	28.94 (23.94 - 33.20)
	Temperature (°C)	19.31 (3.37)	19.19 (16.47 - 22.23)

© 2022 Niu Z et al. *JAMA Network Open.*

eTable 4a. Population characteristics and birth weight z-scores (BWZ) according to individual and neighborhood level stressors

Population characteristics	CES					PSS		
	Low	High	P		Missing	Low	High	P
**N**	229	394			85	411	132	
**BWZ**	-0.11 (1.38)	-0.05 (6.99)	0.51		-0.20 (1.55)	-0.01 (1.91)	-0.17 (1.55)	0.15
**Enrollment timepoint**								
Regular Entry (<20 weeks)	170 (73.91)	291 (73.48)	0.06		43 (50.59)	323 (78.59)	95 (71.97)	<.001
Late Entry (20-30 weeks)	60 (26.09)	105 (26.52)			42 (49.41)	88 (21.41)	37 (28.03)	
**Language preference**								
English	173 (75.22)	250 (63.13)	0.01		62 (72.94)	277 (67.40)	84 (63.64)	<.001
Spanish	56 (24.35)	145 (36.62)			21 (24.71)	134 (32.60)	48 (36.36)	
**Maternal country of origin**								
Latin America	71 (30.87)	152 (38.38)	0.10		0 (0.00)	166 (40.39)	59 (44.70)	<.001
US	107 (46.52)	173 (43.69)			1 (1.18)	215 (52.31)	64 (48.48)	
Others/unknown [1]	52 (22.61)	71 (17.93)			84 (98.82)	30 (7.30)	9 (6.82)	
**Maternal ethnicity**								
Non-Hispanic	145 (63.04)	316 (79.80)	<.001		43 (50.59)	320 (77.86)	100 (75.76)	<.001
Hispanic	74 (32.17)	56 (14.14)			11 (12.94)	87 (21.17)	32 (24.24)	
**Cohabitation status**								
Cohabitate with spouse or partner	133 (57.83)	238 (60.10)	0.74		1 (1.18)	287 (69.83)	85 (64.39)	<.001
Non-cohabitating	52 (22.61)	79 (19.95)			0 (0.00)	95 (23.11)	36 (27.27)	
Missing/decline to respond	45 (19.57)	79 (19.95)			84 (98.82)	29 (7.06)	11 (8.33)	
**Annual household income ($)**								
<15,000	33 (14.35)	76 (19.19)	<.001		10 (11.76)	66 (16.06)	34 (25.76)	<.001
15,000-29,000	54 (23.48)	91 (22.98)			9 (10.59)	110 (26.76)	27 (20.45)	
≥30,000	72 (31.30)	66 (16.67)			6 (7.06)	100 (24.33)	32 (24.24)	
Unknown	60 (26.09)	140 (35.35)			30 (35.29)	131 (31.87)	39 (29.55)	
Missing	11 (4.78)	23 (5.81)			30 (35.29)	4 (0.97)	0 (0.00)	
**Education**								
Below 12th grade	39 (16.96)	111 (28.03)	<.001		19 (22.35)	92 (22.38)	39 (29.55)	<.001
Completed 12th grade	53 (23.04)	122 (30.81)			19 (22.35)	125 (30.41)	33 (25.00)	
Some College	70 (30.43)	94 (23.74)			15 (17.65)	112 (27.25)	37 (28.03)	
College or above	57 (24.78)	46 (11.62)			2 (2.35)	78 (18.98)	23 (17.42)	
Missing	11 (4.78)	23 (5.81)			30 (35.29)	4 (0.97)	0 (0.00)	
**Pre-pregnancy BMI category**								
Normal/underweight	84 (36.52)	120 (30.30)	0.21		25 (29.41)	128 (31.14)	51 (38.64)	<.001
Overweight	67 (29.13)	125 (31.57)			22 (25.88)	137 (33.33)	35 (26.52)	
Obese	77 (33.48)	150 (37.88)			35 (41.18)	146 (35.52)	46 (34.85)	
**Birth order**								
First born	79 (34.35)	112 (28.28)	0.47		1 (1.18)	148 (36.01)	42 (31.82)	<.001
Second born	61 (26.52)	105 (26.52)			0 (0.00)	129 (31.39)	38 (28.79)	
Third or later born	52 (22.61)	114 (28.79)			0 (0.00)	120 (29.20)	47 (35.61)	
Missing	38 (16.52)	65 (16.41)			84 (98.82)	14 (3.41)	5 (3.79)	
**Newborn sex**								
Female	122 (53.04)	199 (50.25)	0.80		44 (51.76)	208 (50.61)	70 (53.03)	0.89
Male	108 (46.96)	197 (49.75)			41 (48.24)	203 (49.39)	62 (46.97)	
**12 weeks preconception**								
$PM_{2.5}$ (ug/m^3)	11.88 (2.29)	11.93 (2.21)	0.84		12.31 (2.11)	11.88 (2.24)	11.75 (2.28)	0.18
PM_{10} (ug/m3)	29.17 (6.85)	29.57 (5.64)	0.41		29.91 (6.12)	29.46 (5.66)	29.03 (5.88)	0.55
NO_2 (ppb)	16.22 (5.75)	18.11 (5.82)	<.0001		17.97 (5.97)	17.27 (5.83)	17.51 (5.95)	0.59
O_3 (ppb)	42.60 (7.66)	41.43 (7.43)	0.06		41.52 (7.44)	42.00 (7.59)	41.65 (7.44)	0.82
**1st trimester**								
$PM_{2.5}$ (ug/m^3)	11.89 (2.25)	11.96 (2.34)	0.70		12.21 (2.45)	11.94 (2.33)	11.73 (2.12)	0.33
PM_{10} (ug/m3)	29.11 (6.28)	29.59 (5.74)	0.33		29.75 (6.24)	29.51 (5.78)	28.89 (6.25)	0.51
NO_2 (ppb)	15.51 (5.61)	17.72 (6.16)	<.001		18.01 (6.43)	16.70 (6.85)	16.79 (5.78)	0.20
O_3 (ppb)	43.75 (7.33)	41.56 (6.84)	<.001		41.15 (7.53)	42.46 (7.13)	42.87 (7.64)	0.21
**2nd trimester**								
$PM_{2.5}$ (ug/m^3)	12.14 (2.19)	12.02 (2.23)	0.53		11.92 (2.29)	12.06 (2.19)	12.18 (2.19)	0.69

© 2022 Niu Z et al. *JAMA Network Open.*

	PM_{10} (ug/m3)	29.35 (6.17)	29.08 (5.74)	0.59		29.24 (5.64)	29.10 (6.71)	29.36 (5.74)	0.91
	NO_2 (ppb)	15.94 (5.75)	16.74 (5.79)	0.10		15.79 (5.98)	16.39 (5.76)	17.00 (5.93)	0.32
	O_3 (ppb)	43.33 (7.53)	42.35 (6.76)	0.09		42.78 (6.63)	42.66 (7.14)	42.85 (7.11)	0.96
**3rd trimester**									
	$PM_{2.5}$ (ug/m^3)	11.99 (2.24)	12.23 (2.39)	0.22		12.28 (2.34)	12.11 (2.38)	12.16 (2.15)	0.83
	PM_{10} (ug/m3)	28.60 (6.39)	28.78 (6.44)	0.75		29.65 (6.05)	28.48 (6.47)	28.84 (6.48)	0.31
	NO_2 (ppb)	16.15 (5.67)	17.14 (6.24)	0.05		15.76 (6.17)	16.83 (5.97)	17.26 (5.53)	0.19
	O_3 (ppb)	42.81 (8.57)	41.76 (7.39)	0.11		43.11 (6.58)	41.95 (7.72)	42.17 (8.72)	0.47
**Whole pregnancy**									
	$PM_{2.5}$ (ug/m^3)	12.01 (1.19)	12.07 (1.48)	0.49		12.13 (1.54)	12.04 (1.13)	12.03 (1.17)	0.76
	PM_{10} (ug/m3)	29.08 (4.78)	29.15 (4.76)	0.86		29.54 (4.58)	29.06 (4.85)	29.08 (4.63)	0.70
	NO_2 (ppb)	15.85 (3.14)	17.19 (2.73)	<.001		16.54 (3.15)	16.64 (2.91)	16.99 (2.85)	0.42
	O_3 (ppb)	43.31 (4.11)	41.94 (3.47)	<.001		42.32 (3.38)	42.39 (3.88)	42.68 (3.68)	0.71

© 2022 Niu Z et al. *JAMA Network Open.*

eTable 4b. Population characteristics and birth weight z-scores (BWZ) according to the joint distribution of individual and neighborhood level stressors

Population characteristics		Joint distribution by PSS and CES				
		Low CES + low PSS	High CES + low PSS	Low CES + high PSS	High CES + high PSS	P
**N**		150	260	49	82	
**BWZ**		-0.05 (1.85)	0.00 (4.99)	-0.20 (1.14)	-0.15 (1.71)	0.44
**Enrollment timepoint**						
	Regular Entry (<20 weeks)	116 (77.33)	207 (79.62)	39 (79.59)	56 (68.29)	<.001
	Late Entry (20-30 weeks)	34 (22.67)	53 (20.38)	10 (20.41)	26 (31.71)	
**Language preference**						
	English	113 (75.33)	164 (63.08)	35 (71.43)	49 (59.76)	<.001
	Spanish	37 (24.67)	96 (36.92)	14 (28.57)	33 (40.24)	
**Maternal country of origin**						
	Latin America	51 (34.00)	36 (13.85)	18 (36.73)	14 (17.07)	<.001
	US	42 (28.00)	104 (40.00)	12 (24.49)	23 (28.05)	
	Others/unknown [1]	53 (35.33)	112 (43.08)	18 (36.73)	41 (50.00)	
**Maternal ethnicity**						
	Non-Hispanic	51 (34.00)	36 (13.85)	18 (36.73)	14 (17.07)	<.001
	Hispanic	96 (64.00)	223 (85.77)	31 (63.27)	68 (82.93)	
	Missing					
**Cohabitation status**						
	Cohabitate with spouse or partner	104 (69.33)	182 (70.00)	29 (59.18)	55 (67.07)	<.001
	Non-cohabitating	36 (24.00)	59 (22.69)	16 (32.65)	20 (24.39)	
	Missing/decline to respond	10 (6.67)	19 (7.31)	4 (8.16)	7 (8.54)	
**Annual household income ($)**						
	<15,000	16 (10.67)	50 (19.23)	13 (26.53)	20 (24.39)	<.001
	15,000-29,000	40 (26.67)	69 (26.54)	10 (20.41)	17 (20.73)	
	≥30,000	52 (34.67)	48 (18.46)	16 (32.65)	16 (19.51)	
	Unknown	39 (26.00)	92 (35.38)	10 (20.41)	29 (35.37)	
	Missing	3 (2.00)	1 (0.38)	0 (0.00)	0 (0.00)	
**Education**						
	Below 12th grade	23 (15.33)	69 (26.54)	10 (20.41)	29 (35.37)	<.001
	Completed 12th grade	38 (25.33)	86 (33.08)	10 (20.41)	22 (26.83)	
	Some College	41 (27.33)	71 (27.31)	18 (36.73)	19 (23.17)	
	College or above	45 (30.00)	33 (12.69)	11 (22.45)	12 (14.63)	
	Missing	3 (2.00)	1 (0.38)	0 (0.00)	0 (0.00)	
**Pre-pregnancy BMI category**						
	Normal/underweight	49 (32.67)	79 (30.38)	23 (46.94)	28 (34.15)	0.01
	Overweight	52 (34.67)	84 (32.31)	9 (18.37)	25 (30.49)	
	Obese	49 (32.67)	97 (37.31)	17 (34.69)	29 (35.37)	
**Birth order**						
	First born	62 (41.33)	86 (33.08)	17 (34.69)	25 (30.49)	<.001
	Second born	47 (31.33)	81 (31.15)	14 (28.57)	24 (29.27)	
	Third or later born	35 (23.33)	85 (32.69)	17 (34.69)	29 (35.37)	
	Missing	6 (4.00)	8 (3.08)	1 (2.04)	4 (4.88)	
**Newborn sex**						
	Female	76 (50.67)	131 (50.38)	29 (59.18)	41 (50.00)	0.85
	Male	74 (49.33)	129 (49.62)	20 (40.82)	41 (50.00)	
**12 weeks preconception**						
	$PM_{2.5}$ (ug/m^3)	11.84 (2.11)	11.91 (2.25)	11.58 (2.85)	11.85 (2.42)	0.83
	PM_{10} (ug/m3)	29.13 (5.85)	29.64 (5.55)	28.65 (5.95)	29.27 (5.86)	0.65
	NO_2 (ppb)	15.94 (5.68)	18.03 (5.79)	16.34 (5.25)	18.22 (6.18)	0.00
	O_3 (ppb)	42.93 (7.81)	41.45 (7.43)	43.12 (7.31)	40.75 (7.43)	0.08
**1st trimester**						
	$PM_{2.5}$ (ug/m^3)	11.97 (2.32)	11.92 (2.34)	11.87 (2.25)	11.64 (2.25)	0.75
	PM_{10} (ug/m3)	29.58 (6.48)	29.46 (5.41)	28.40 (6.84)	29.20 (5.88)	0.65
	NO_2 (ppb)	15.57 (5.85)	17.36 (6.87)	15.79 (5.47)	17.39 (5.91)	0.01
	O_3 (ppb)	43.85 (7.24)	41.65 (6.96)	43.88 (8.66)	42.26 (5.73)	0.01

© 2022 Niu Z et al. *JAMA Network Open.*

**2nd trimester**						
	$PM_{2.5}$ (ug/m^3)	12.17 (2.12)	12.00 (2.22)	12.17 (2.47)	12.18 (2.72)	0.83
	PM_{10} (ug/m3)	29.34 (6.16)	28.97 (5.93)	29.81 (7.13)	29.09 (4.76)	0.80
	NO_2 (ppb)	16.17 (5.73)	16.53 (5.69)	16.79 (5.88)	17.12 (5.99)	0.67
	O_3 (ppb)	42.91 (7.66)	42.51 (6.83)	44.18 (7.42)	42.06 (6.83)	0.38
**3rd trimester**						
	$PM_{2.5}$ (ug/m^3)	11.89 (2.23)	12.24 (2.47)	12.12 (2.12)	12.18 (2.17)	0.54
	PM_{10} (ug/m3)	28.21 (6.59)	28.64 (6.68)	29.46 (7.29)	28.47 (5.97)	0.70
	NO_2 (ppb)	16.01 (5.50)	17.31 (6.19)	17.09 (5.85)	17.37 (5.37)	0.16
	O_3 (ppb)	42.54 (8.12)	41.62 (7.48)	43.27 (16.51)	41.51 (7.44)	0.42
**Whole pregnancy**						
	$PM_{2.5}$ (ug/m^3)	12.01 (1.16)	12.06 (1.11)	12.06 (1.45)	12.00 (9.97)	0.98
	PM_{10} (ug/m3)	29.13 (4.81)	29.02 (4.87)	29.28 (5.34)	28.95 (4.17)	0.98
	NO_2 (ppb)	15.91 (3.81)	17.06 (2.76)	16.52 (3.09)	17.27 (2.63)	<.001
	O_3 (ppb)	43.11 (4.49)	41.97 (3.74)	43.79 (4.36)	42.02 (3.44)	<.001

© 2022 Niu Z et al. *JAMA Network Open.*

eTable 5. Average weekly effect estimation of air pollution exposure in identified sensitive windows on birth weight

Air pollutant	Subgroup	Sensitive window (gestational week)[a]	Average difference in birthweight (gram, 95% CI) [b]
PM2.5	Overall	14-22	-9.5 (-10.4, -8.6)
	High-PSS	10-14	-15.4 (-18.4, -12.4)
	High-CES	10-22	-15.4 (-16.2, -14.5)
	High-PSS/High-CES	4-20	-34.0 (-35.7, -32.4)
PM10	High-PSS	9-12	-19.2 (-28.6, -9.9)
	High-CES	5-17	-16.7 (-17.6, -15.8)
	High-CES	25-32	18.5 (16.6, 20.3)
	Low-PSS/Low-CES	21-24	-17.9 (-22.2, -13.6)
	Low-PSS/Low-CES	31-32	-23.9 (-35.0, -12.8)
	Low-PSS/High-CES	8-10	-14.2 (-18.8, -9.7)
	High-PSS/High-CES	9-14	-39.4 (-45.4, -33.4)
NO2	Overall	9-14	-13.5 (-15.6, -11.5)
	High-PSS	8-9	-19.2 (-28.6, -9.9)
	High-PSS/Low-CES	5-8	-35.5 (-43.6, -27.4)
	High-PSS/Low-CES	31	-43.5 (-84.8, -2.3)
	High-PSS/High-CES	9-14	-40.4 (-47.4, -33.3)
	High-PSS/High-CES	23-26	63.2 (47.8, 78.5)
	High-PSS/High-CES	33-36	-117.6 (-141.0, -94.2)

Note: a: sensitive windows were identified from DLM models (visualized in Figures 1-4). All model adjusted for weekly temperature, maternal age, education, maternal pre-pregnancy body mass index, maternal ethnicity and nativity, marital status, and birth order. b: difference in birthweight was estimated assuming 1 unit of BWZ=450 grams of birthweight. Effect estimation was based on per IQR increases in each air pollutant (i.e., 4 $\mu g/m^3$, 12 $\mu g/m^3$, 11 ppb, and 15 ppb for $PM_{2.5}$, and PM_{10}, NO_2, and O_3, respectively).

© 2022 Niu Z et al. *JAMA Network Open.*

eTable 6: Population characteristics and birth weight z-scores (BWZ) according to preterm birth status among all participants in the MADRES cohort (2015-2021)

Population characteristics		PTB		
		No	Yes	P
**N**		628	72	
**Overall**		-0.07±1.73	0.18±1.48	0.04
**Enrollment timepoint**				
	Regular Entry (<20 weeks)	461 (73.41)	58 (80.56)	0.19
	Late Entry (20-30 weeks)	167 (26.59)	14 (19.44)	
**Language preference**				
	English	423 (67.36)	49 (68.06)	0.89
	Spanish	203 (32.32)	23 (31.94)	
**Maternal country of origin**				
	Latin America	225 (35.83)	20 (27.78)	0.40
	US	280 (44.59)	36 (50.00)	
	Others/unknown [1]	123 (19.59)	16 (22.22)	
**Maternal ethnicity**				
	Non-Hispanic	130 (20.70)	10 (13.89)	0.29
	Hispanic	463 (73.73)	56 (77.78)	
	Missing			
**Cohabitation status**				
	Cohabitate with spouse or partner	373 (59.39)	40 (55.56)	0.81
	Non-cohabitating	131 (20.86)	17 (23.61)	
	Missing/decline to respond	124 (19.75)	15 (20.83)	
**Annual household income ($)**				
	<15,000	110 (17.52)	23 (31.94)	**0.02**
	15,000-29,000	146 (23.25)	17 (23.61)	
	≥30,000	138 (21.97)	9 (12.50)	
	Unknown	200 (31.85)	17 (23.61)	
	Missing	34 (5.41)	6 (8.33)	
**Education**				
	Below 12th grade	150 (23.89)	19 (26.39)	**0.04**
	Completed 12th grade	177 (28.18)	28 (38.89)	
	Some College	164 (26.11)	13 (18.06)	
	College or above	103 (16.40)	5 (6.94)	
	Missing	34 (5.41)	7 (9.72)	
**Pre-pregnancy BMI category**				
	Normal/underweight	204 (32.48)	21 (29.17)	0.83
	Overweight	194 (30.89)	22 (30.56)	
	Obese	227 (36.15)	29 (40.28)	
**Birth order**				
	First born	191 (30.41)	17 (23.61)	0.51
	Second born	167 (26.59)	19 (26.39)	
	Third or later born	167 (26.59)	20 (27.78)	
	Missing	103 (16.40)	16 (22.22)	
**Newborn sex**				
	Female	322 (51.27)	31 (43.06)	0.19
	Male	306 (48.73)	41 (56.94)	
**12 weeks preconception**				
	$PM_{2.5}$ (ug/m^3)	11.91±2.21	11.84±1.76	0.81
	PM_{10} (ug/m3)	29.43±5.76	29.97±5.48	0.45
	NO_2 (ppb)	17.41±5.86	16.01±5.25	**0.05**
	O_3 (ppb)	41.86±7.53	44.19±8.33	**0.01**
**1st trimester**				
	$PM_{2.5}$ (ug/m^3)	11.93±2.34	12.14±2.11	0.47
	PM_{10} (ug/m3)	29.41±5.94	29.83±5.97	0.57
	NO_2 (ppb)	16.89±6.95	17.24±5.54	0.65
	O_3 (ppb)	42.37±7.11	42.56±7.61	0.83

© 2022 Niu Z et al. *JAMA Network Open.*

**2nd trimester**				
	$PM_{2.5}$ (ug/m^3)	12.06±2.26	12.31±2.62	0.39
	PM_{10} (ug/m3)	29.17±5.99	29.67±8.55	0.52
	NO_2 (ppb)	16.44±5.79	17.96±6.15	**0.04**
	O_3 (ppb)	42.71±7.45	40.90±7.35	**0.04**
**3rd trimester**				
	$PM_{2.5}$ (ug/m^3)	12.15±2.32	12.17±3.68	0.92
	PM_{10} (ug/m3)	28.71±6.42	28.77±6.87	0.95
	NO_2 (ppb)	16.78±5.92	16.90±6.23	0.87
	O_3 (ppb)	42.15±7.82	41.47±8.12	0.49
**Whole pregnancy**				
	$PM_{2.5}$ (ug/m^3)	12.05±1.12	12.22±1.43	0.23
	PM_{10} (ug/m3)	29.13±4.76	29.52±5.52	0.51
	NO_2 (ppb)	16.70±2.92	17.48±3.32	**0.04**
	O_3 (ppb)	42.44±3.77	41.64±3.26	0.08

© 2022 Niu Z et al. *JAMA Network Open.*

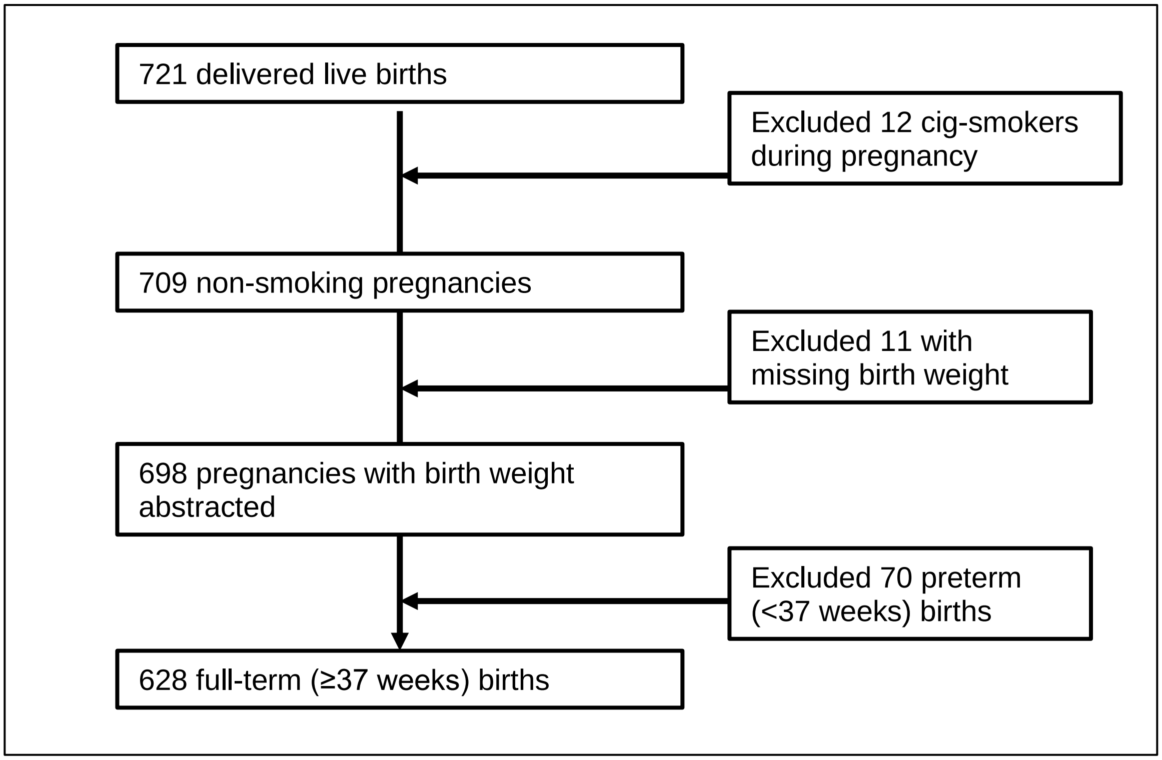

eFigure 1. Flow chart of participants included in this analysis from the MADRES cohort.

© 2022 Niu Z et al. *JAMA Network Open.*

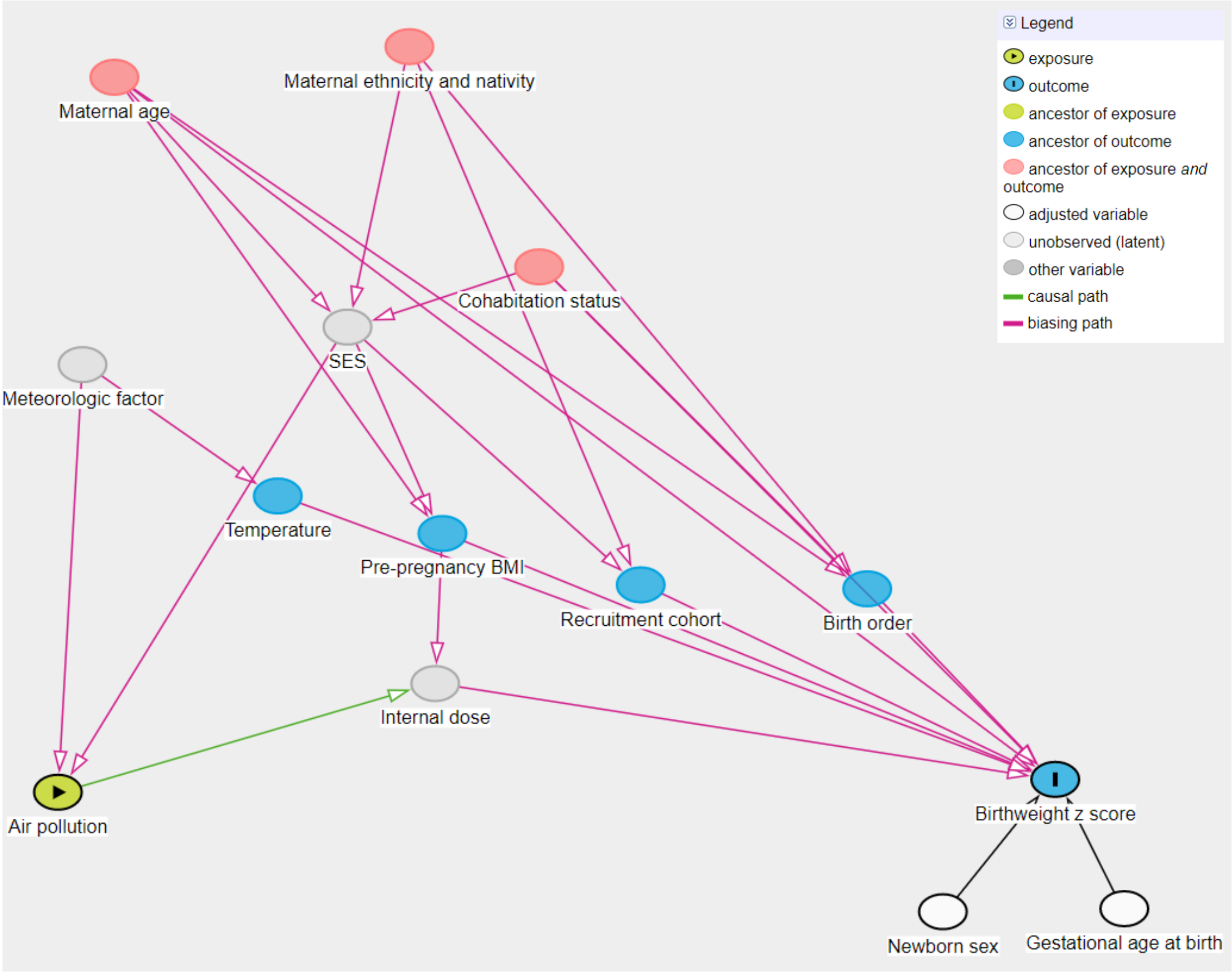

eFigure 2. Theoretical causal structure underlying the association of air pollution exposure with BWZ.

© 2022 Niu Z et al. *JAMA Network Open.*

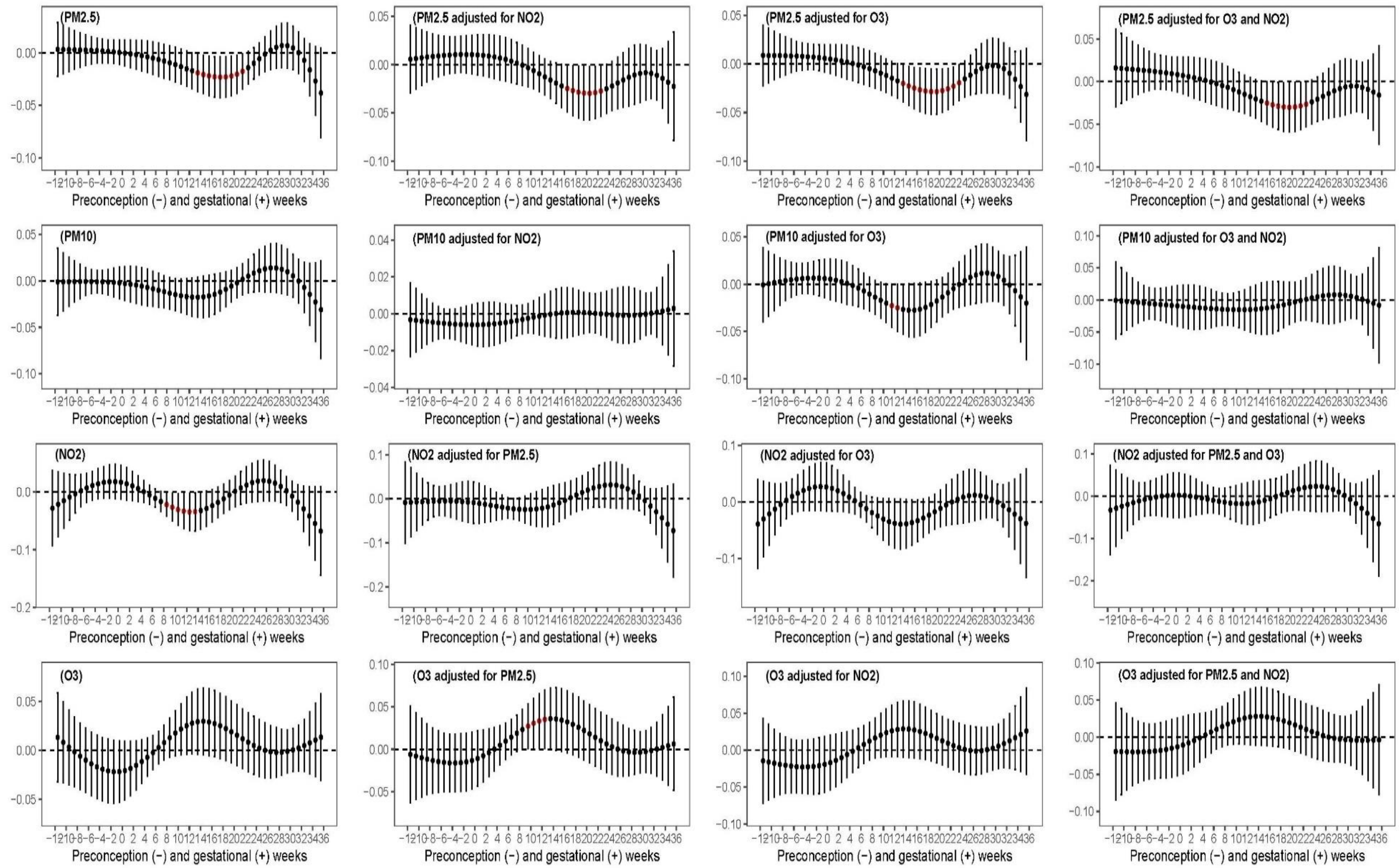

eFigure 3. Sensitivity analysis with multiple pollutants in DLM. Associations were adjusted for weekly temperature, maternal age, education, pre-pregnancy body mass index, maternal ethnicity and nativity, marital status, and birth order. Effect estimation was based on per IQR increases in each air pollutant (i.e., 4 $\mu g/m^3$, 12 $\mu g/m^3$, 11 ppb, and 15 ppb for $PM_{2.5}$, and PM_{10}, NO_2, and O_3, respectively).

© 2022 Niu Z et al. *JAMA Network Open.*

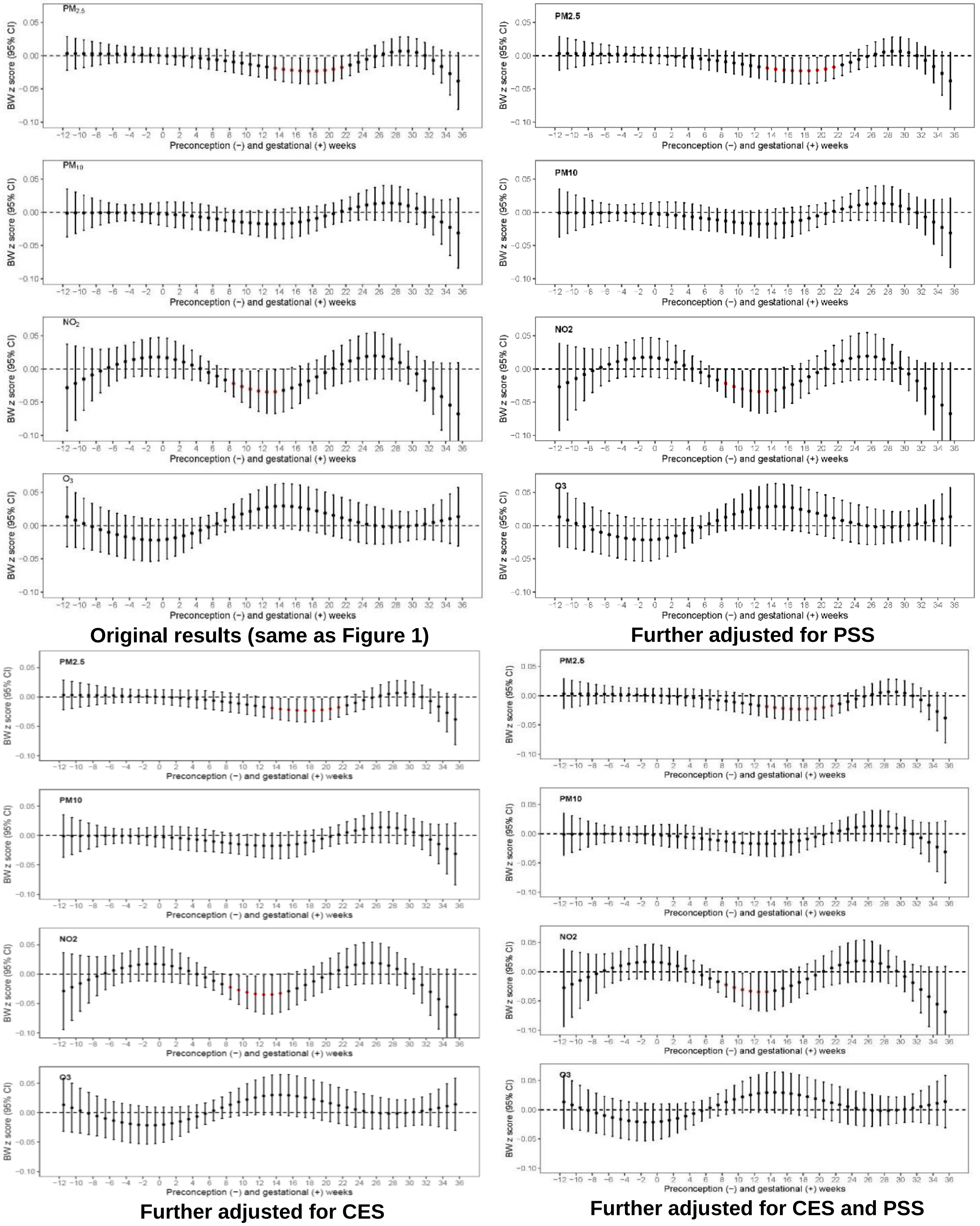

eFigure 4. Sensitivity analysis adjusting for CES, PSS, or both. Associations were further adjusted for weekly temperature, maternal age, education, pre-pregnancy body mass index, maternal ethnicity and nativity, marital status, and birth order. Effect estimation was based on per IQR increases in each air pollutant (i.e., 4 $\mu g/m^3$, 12 $\mu g/m^3$, 11 ppb, and 15 ppb for $PM_{2.5}$, and PM_{10}, NO_2, and O_3, respectively).

© 2022 Niu Z et al. *JAMA Network Open.*

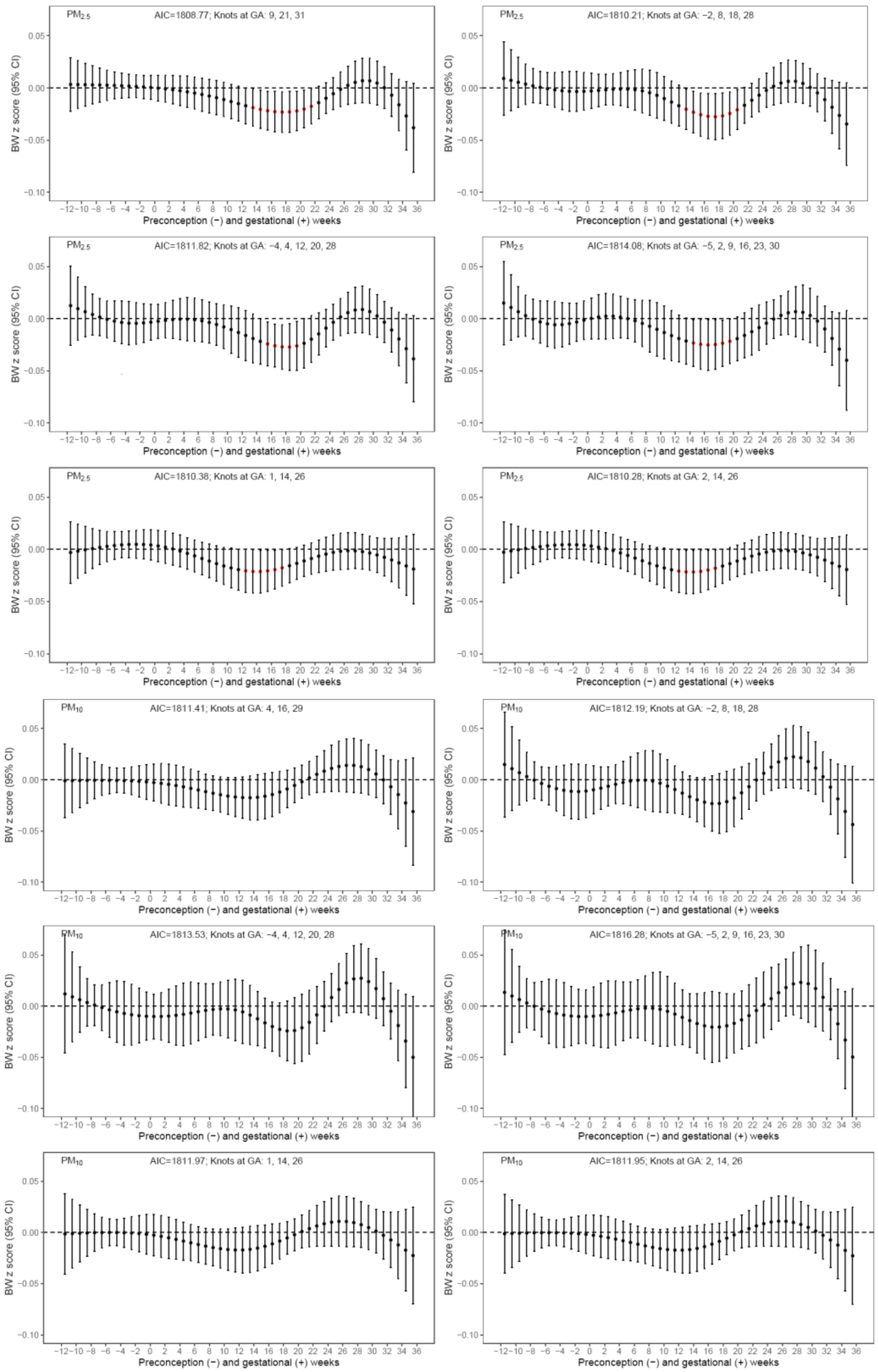

eFigure 5a. Sensitivity analysis with various knots placement for $PM_{2.5}$ and PM_{10}. Model fitting and knots selection were based on minimized AIC. Associations were adjusted for weekly temperature, maternal age, education, pre-pregnancy body mass index, maternal ethnicity and nativity, marital status, and birth order. Effect estimation was based on per IQR increases in each air pollutant (i.e., 4 $\mu g/m^3$, 12 $\mu g/m^3$ for $PM_{2.5}$ and PM_{10}, respectively).

© 2022 Niu Z et al. *JAMA Network Open.*

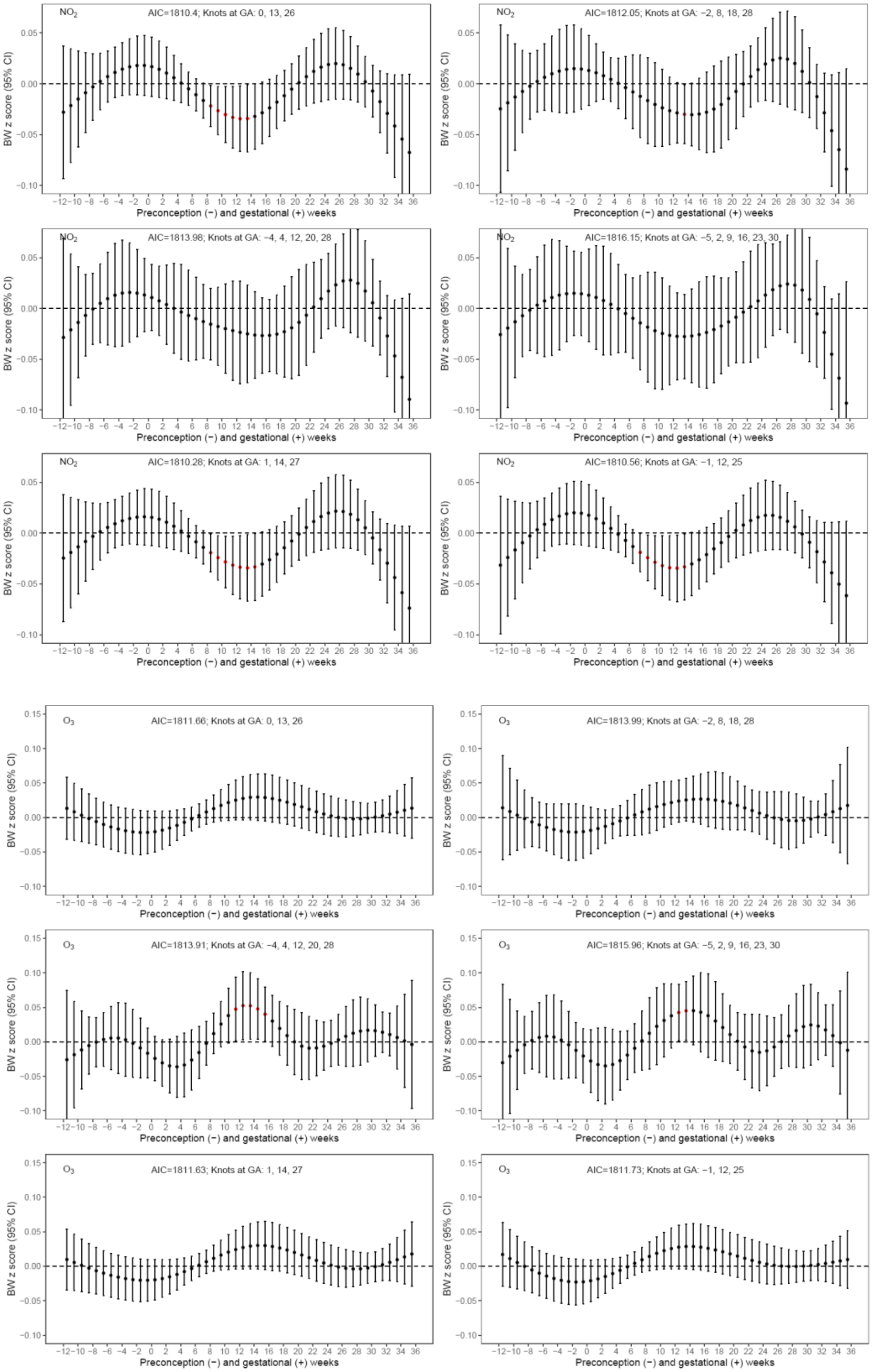

eFigure 5b. Sensitivity analysis with various knots placement for NO_2 and O_3. Model fitting and knots selection were based on minimized AIC. Associations were adjusted for weekly temperature, maternal age, education, pre-pregnancy body mass index, maternal ethnicity and nativity, marital status, and birth order. Effect estimation was based on per IQR increases in each air pollutant (i.e., 11 ppb, and 15 ppb for NO_2 and O_3, respectively).

© 2022 Niu Z et al. *JAMA Network Open.*

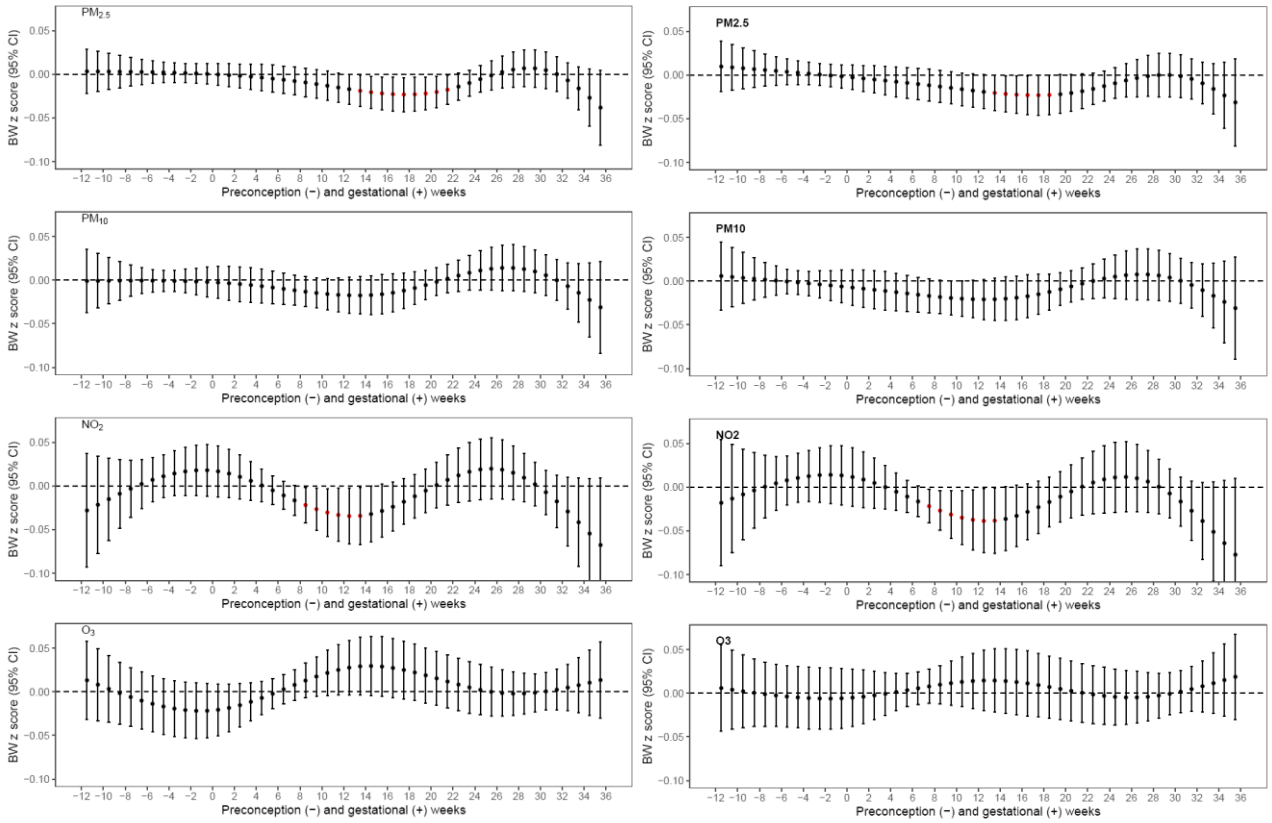

Original results

Complete case analysis results

eFigure 6. Sensitivity analysis with complete cases. In the complete case analysis, 114 participants were removed due to missingness of ethnicity (n=35), cohabitating status (n=103), and education (n=34). Associations were adjusted for weekly temperature, maternal age, education, pre-pregnancy body mass index, maternal ethnicity and nativity, marital status, and birth order. Effect estimation was based on per IQR increases in each air pollutant (i.e., 4 $\mu g/m^3$, 12 $\mu g/m^3$, 11 ppb, and 15 ppb for $PM_{2.5}$, and PM_{10}, NO_2, and O_3, respectively).

© 2022 Niu Z et al. *JAMA Network Open.*
